# Facile Syntheses and Molecular-Docking of Novel Substituted 3,4-Dimethyl-1*H*-pyrrole-2-carboxamide/carbohydrazide Analogues with Antimicrobial and Antifungal Properties

**DOI:** 10.3390/molecules23040875

**Published:** 2018-04-11

**Authors:** Jitendra D. Bhosale, Rajesh Dabur, Gopal P. Jadhav, R. S. Bendre

**Affiliations:** 1 School of Chemical Sciences, North Maharashtra University, Jalgaon 425001, India; bhosalejd@gmail.com; 2 Department of Biochemistry, Maharshi Dayanand University, Rohtak 124001, India; rajeshdabur@yahoo.com; 3 School of Medicine, Department of clinical & translational sciences, Creighton University, Omaha, NE 68178, USA

**Keywords:** carboxamide, carbohydrazine, antibacterial, antifungal, molecular docking, Schiff base, NMR, IR

## Abstract

The article describes the use of facile one-pot, high-yielding reactions to synthesize substituted 3,4-dimethyl-1*H*-pyrrole-2-carboxamides **3a**–**m** and carbohydrazide analogues **5a**–**l** as potential antifungal and antimicrobial agents. The structural identity and purity of the synthesized compounds were assigned based on appropriate spectroscopic techniques. Synthesized compounds were assessed in vitro for antifungal and antibacterial activity. The compounds **5h**, **5i** and **5j** were found to be the most potent against *Aspergillus* *fumigatus*, with MIC values of 0.039 mg/mL. The compound **5f** bearing a 2, 6-dichloro group on the phenyl ring was found to be the most active broad spectrum antibacterial agent with a MIC value of 0.039 mg/mL. The mode of action of the most promising antifungal compounds (one representative from each series; **3j** and **5h**) was established by their molecular docking with the active site of sterol 14α-demethylase. Molecular docking studies revealed a highly spontaneous binding ability of the tested compounds in the access channel away from catalytic heme iron of the enzyme, which suggested that the tested compounds inhibit this enzyme and would avoid heme iron-related deleterious side effects observed with many existing antifungal compounds.

## 1. Introduction

**Figure 1:**
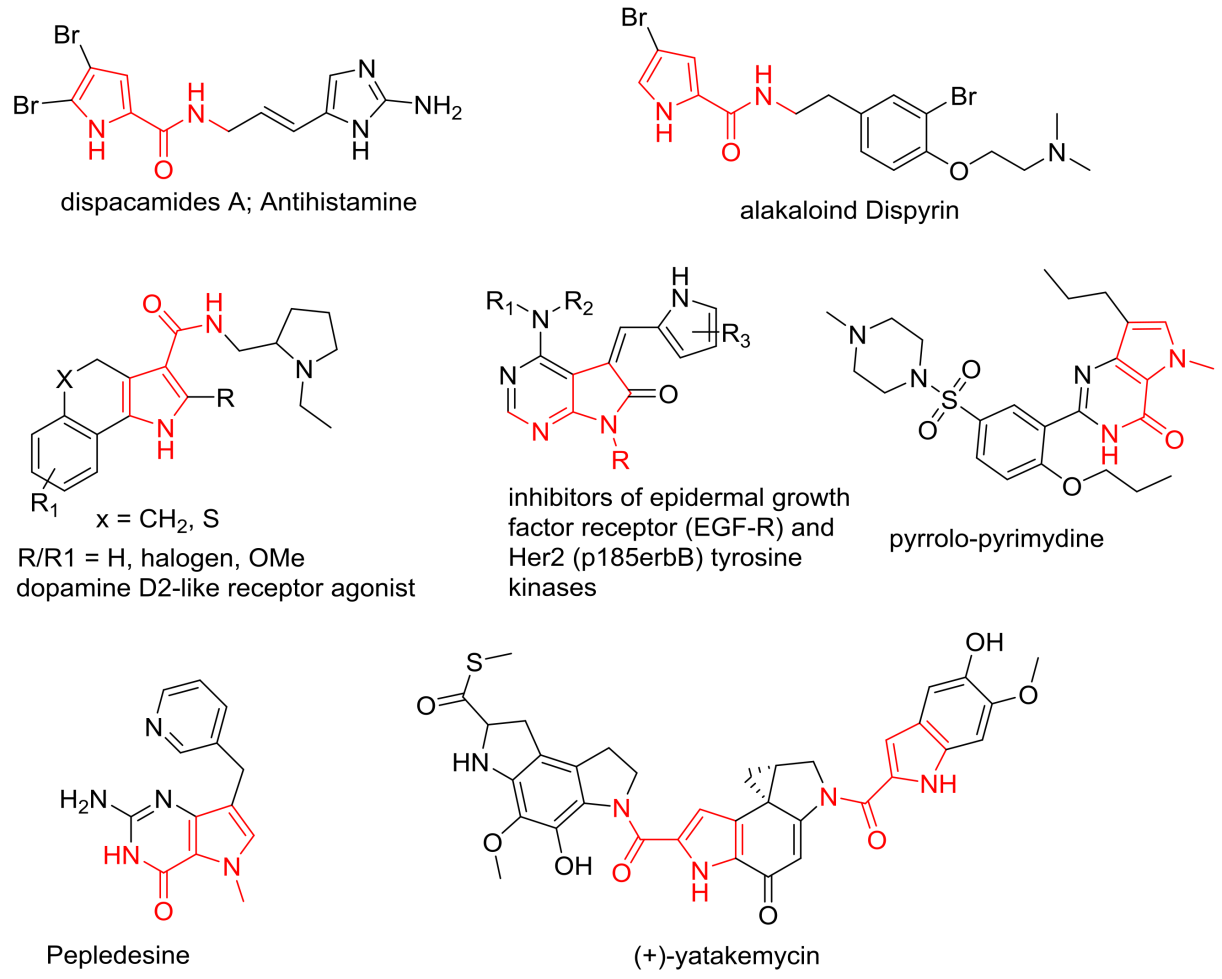
Pyrrole-caboxamide skeletons (red) in diverse biologically active compounds.

The pyrrole carboxamide skeleton is an important and unique class of heterocyclic compounds which has yet to be well explored [1,2]. Since carboxamides are neutral, stable and have both hydrogen-bonds accepting and donating properties, they have been incorporated into the broad range of synthetic molecules, agrochemicals and pharmaceuticals. Numerous marine-derived novel compounds [3] containing an integral carboxamide motif as highlighted in Figure 1 exhibit varied biological activities that are important for potential drug development, e.g., dispyrin, a bromopyrrole alkaloid (antagonist of α_2A_, H_2_ and H_3_ histamine receptors), dispacamide-A (anti-histaminic), sceptrin (antiviral), agelastatin-A, yatakemycin, (antitumor), storniamide-A (antibacterial), hymenialdisine (kinase inhibitor), distamycin and netropsin (DNA minor groove binders) [4,5,6,7,8,9,10,11]. Synthetic pyrrole carboxamides show a wide range of pharmacological properties, including tyrosine kinase inhibition. They bind to dopamine-D_2_-like receptors and modulate protein kinase activity. The pyrrole carboxamide motif is part of many blockbuster drugs like the calcium salt of atorvastatin (a hypolipidemic agent), lisinopril (an inhibitor of angiotensin-converting enzyme), valsartan (angiotensin-II receptor blocker), diltiazem (calcium channel blocker), istamycin and pyrronamycin A & B (used as antibiotics) [12,13,14,15,16,17,18,19,20].

Another structural scaffold that involves hydrazide nitrogens double bonded to carbon atoms (-C=N-NH-), has attracted the attention of many pharmaceutical and academic researchers due to its ability to map a spectrum of biological activity. The range of activities that Schiff base hydrazides display, which include anticancer, antimicrobial effects, etc., make them important organic scaffolds [21,22,23]. Encouraged by the above demonstrated potentials of pyrrole carboxamides and carbohydrazide Schiff bases as drug scaffolds, their biological diversity and as a part of our ongoing research on novel derivatives of the 3,4-dimethylpyrrole molecule [24], we employed a structure activity relationship approach to design new analogues. To this end, we have synthesized some novel 3,4-dimethylpyrrole carboxamide and carbohydrazide analogues as potential antimicrobial and antifungal agents. These analogues retain their novelty, although many synthetic methods to prepare the 3,4-dimethylpyrrole substituted Schiff base and carboxamide appeared to meet our requirements [25]. The best active compounds were subjected to a molecular docking study in the target protein sterol 14α-demethylase (CYP51B) from the fungus *Aspergillus fumigatus* to explore their mode of action [26].

## 2. Results and Discussion

### 2.1. Chemistry

**Scheme 1:**
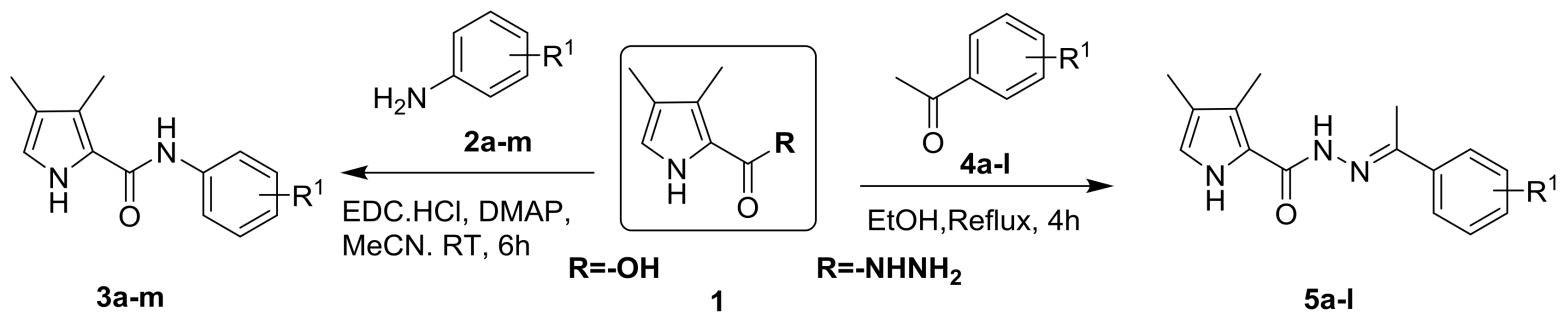
Facile synthesis of substituted 3,4-dimethyl-1*H*-pyrrole-2-caboxamides **3a**–**m** and carbohydrazides **5a**–**l**.

**Figure 2:**
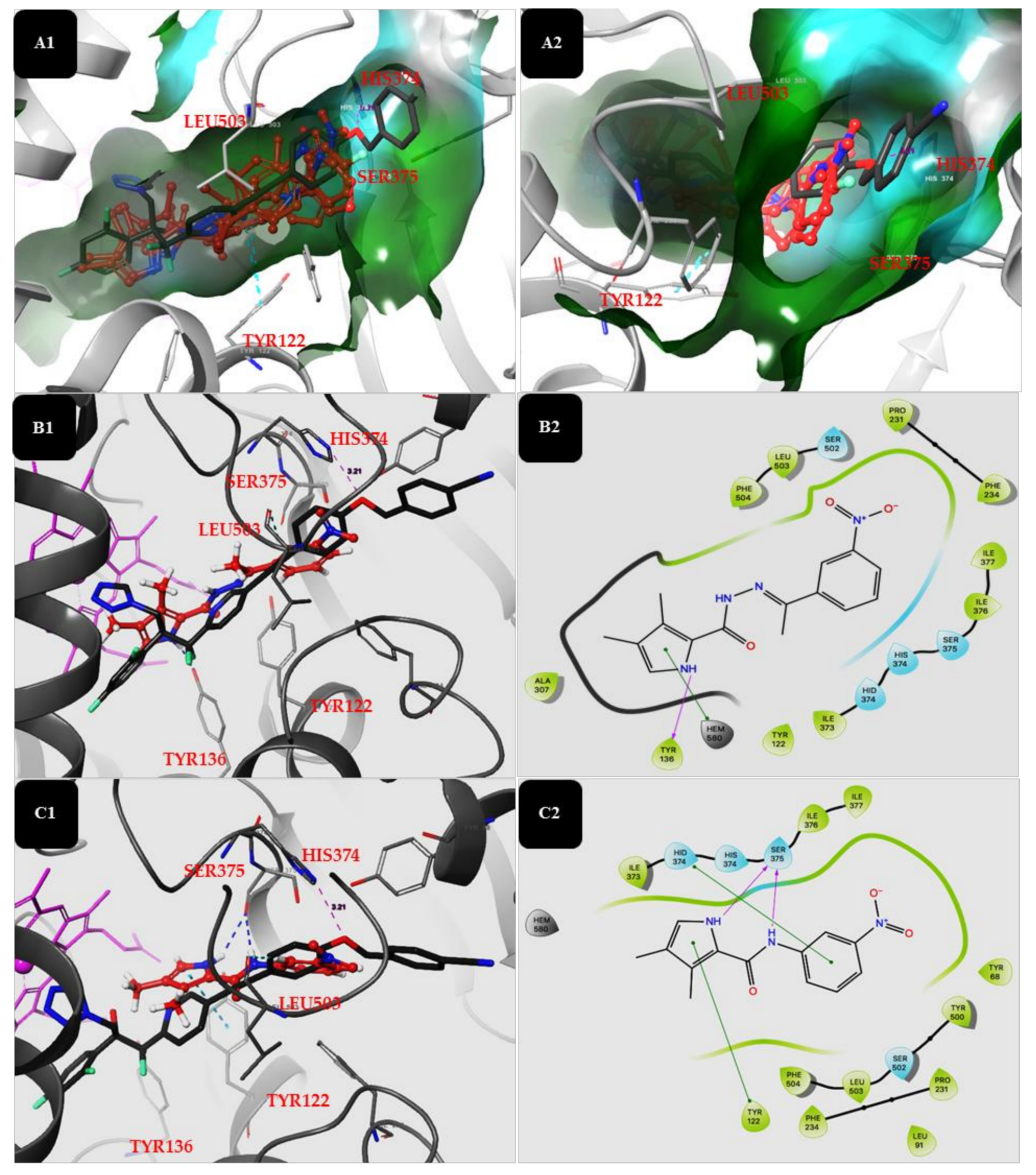
The representation of the binding patterns into the active site of CYP51B for the VT-1598, used as the reference antifungal drug and for the novel compounds; Side view (**A1**) and front view (**A2**) of overlay docking poses of novel potent compounds (red) and VT-1598 (black); Docking poses of the reference compound VT-1598 (black) and compound **5h** (red) in the active site of the CYP51B sterol 14α-demethylase of *A. fumigatus* (**B1**), which showed the highest inhibition capacity and its interacting residues (**B2**); Docking poses of the reference compound VT-1598 (black) and compound **3e** (red) in the active site of the CYP51B sterol 14α-demethylase of *A. fumigatus* (**C1**), which showed the highest inhibition capacity and its interacting residues (**C2**).

The search for precise and robust synthetic routes as part of our continued efforts to develop potent antibacterial compounds motivated us to develop a facile synthesis of these bioactive scaffold- containing analogues in one-pot protocols. Here we envisioned that parallel syntheses of 2,4-dimethylpyrrole carboxamide and carbohydrazide derivatives having various substituents on aryl adducts could be achieved in higher yields. As discussed in Figure 2 and the Experimental Section a total of 25 compounds were synthesized in high yields between 72% and 94%. Simple coupling reactions between 3,4-dimethyl-1*H*-pyrrole-2-carboxylic acid and substituted anilines **2a**–**m** using 1-(3-dimethylaminopropyl)-3-ethylcarbodiimide (EDC) hydrochloride salt and dimethylamino- pyridine (DMAP) in acetonitrile as solvent at room temperature provided the desired carboxamide analogues **3a**–**m**, whereas, a library of novel substituted Schiff base analogues **5a**–**l** of 3,4-dimethyl-pyrrole was developed by condensation of the nucleophile 3,4-dimethyl-1*H*-pyrrole-2-carbohydrazide with the variously substituted acetophenones **4a**–**l**. The synthetic scheme is shown in Scheme 1. Notably, here one-pot reactions provided an efficient method for the flexible and rapid synthesis of the target novel analogues.

All the synthesized compounds were characterized for their structural identity and chemical purity by physical and spectroscopic methods. The infrared (IR) spectra of the analogues **3a**–**m** and **5a**–**l** showed characteristic N-H stretching close to the carbonyl group in the 3434–3352 cm^−1^ region, the N-H bands of the pyrrole rings were confirmed in the 3247–3399 cm^−1^ region, amide C=O stretching at 1625–1686 cm^−1^, C=C absorptions between 1455 and 1463 cm^−1^ and typical -C=N stretch absorptions of carbohydrazide analogues **5a**–**l** were observed in the 1500–1527 cm^−1^ region. ^1^H-NMR spectra of all the derivatives showed the N-H proton of the pyrrole ring as a singlet (*s*) between 7.45 and 11.30 ppm, -OCH_3_ protons resonated as a singlet in the 3.80–3.92 ppm range, while other aromatic protons appeared in the expected region between 6.56 and 8.60 ppm. The presence of a carbonyl group on one side and an imide group on other side make the central nitrogen-bearing proton in carbohydrazide less accessible. Thus, the typical de-shielding effect was observed in the -CON(*H*)-N= protons from the carbohydrazide analogues **5a**–**l**, as a singlet in the range of 11.06–11.20 ppm as compared to -CON*H*- protons from the carboxamide analogues **3a**–**m**, which appear as a singlet in the 9.06–11.06 ppm range. The ^13^C-NMR spectra of all the derivatives showed carbon values in the expected regions, while the mass spectra confirmed M^+^ peaks in agreement with the calculated molecular weights of the target compounds as shown in Supplementary data of active compounds. 

### 2.2. Antibacterial Activity

All the novel compounds from both series, the carboxamides **3a**–**m** and Schiff base carbohydrazides **5a**–**l**, were screened for in vitro antibacterial activity against *Pseudomonas aeruginosa, Escherichia coli*, *Klebsiella pneumoniae*, *Salmonella typhi* and *Bacillus subtilis*. Methods, as recommended by the National Committee for Clinical Laboratory Standards (NCCLS), were used to determine the corresponding minimum inhibitory concentration (MIC) values [27,28,29,30]. Stock solutions were prepared in dimethyl sulfoxide (DMSO) and DMSO alone served as solvent control.

As presented in Table 1 the in vitro antibacterial activity profiles indicated that all compounds displayed antimicrobial activities in a range of 0.078–2.5 mg/mL as compared to standard tetracycline (MIC range of 0.00031–0.01 mg/mL). This decent antibacterial activity was retained in compounds bearing electron withdrawing *p*-nitro groups on the phenyl ring (**3j**, **3k** and **5i**). The *o*-dinitro aromatic substitution in the carboxamide series (**3k**, MIC 0.078 mg/mL against *E. coli*) and *o*-dichloro aromatic substitution in the carbohydrazide series (**5f**, MICs 0.078 mg/mL against *P. aeruginosa*, *K. pneumoniae* and 0.039 mg/mL against *S. typhi*) were found to impart potent antibacterial activity. Some compounds among the synthesized derivatives were found to be moderately potent antibacterials, with MIC values in the range of 0.312–0.625 mg/mL as shown in Table 1.

### 2.3. Antifungal Activity

All newly synthesized compounds were evaluated for their in vitro antifungal activity on three strains belonging to the *Aspergillus* genus [31,32]. The minimal inhibitory concentration (MIC), defined as a concentration of tested compound which causes complete growth inhibition, was determined by means of the broth dilution technique (Table 2).

Fluconazole and amphotericin-B were chosen as reference antifungal drugs. Among *Aspergillus*, *A. fumigatus* turned out to be sensitive to the tested derivatives, showing susceptibility to three compounds (**3e**, **3h**, and **3k**) from the carboxamide series with MIC values of 0.156 mg/mL (10 times lower than reference fluconazole) and three compounds (**5h**, **5i** and **5j**) from the Schiff base hydrazide series with MIC values of 0.039 mg/mL (two times less potent to the reference fluconazole). A good activity profile was also observed against other *Aspergillus* for some compounds, i.e., compounds **5b**, **5e**, and **5g** showed MIC values of 0.078 mg/mL against *Aspergillus niger*; compounds **3j** and **3l**, and **5c** showed MICs of 0.156 mg/mL against *Aspergillus flavus.* The structure-activity analysis revealed that among the 3,4-dimethyl-1*H*-pyrrole-2-carbohydrazide analogues **5a**–**l**, compounds **5h**, and **5i** bearing *o-* or *p-*nitrophenyl substitution and **5j** with a *p-*hydroxyaromatic ring were observed as the most active compounds against *A. fumigatus*. On the other hand, in carbohydrazide analogues, *p-*methyl, *p-*chloro and *p-*bromo aromatic substitutions demonstrated decent antifungal activity whereas, *p-*fluoro substitution resulted in diminished antifungal activity against *A. niger*. In the series of 3,4-dimethyl-1H-pyrrole-2-carboxamides **3a**–**m** *p-*fluoro and *m-*nitro aromatic substituents retained antifungal activity against *A. fumigatus*.

### 2.4. Molecular Docking

Molecular docking is an effective and reliable tool able to locate the probable binding interactions of ligands with their target proteins [33,34]. Recently, small chemical pharmacophores have been reported to inhibit cytochrome P450 sterol 14α-demethylase in a wide spectrum of fungal species [35,36,37]. To understand the mechanism of action and antifungal activity of our synthesized analogues, molecular docking studies were employed using the crystal structure of sterol 14α-demethylase (CYP51B, Protein Data Bank; pdb 5frb)) from a pathogenic filamentous fungus *A. fumigatus* [26,38]. The accuracy and appropriate settings of the docking method were validated by re-docking of VT-1598, the co-crystallized ligand with CYP51B in its active site. The root means square deviation (RMSD) value of 0.128 Å was observed between the top scored predicted conformation and the observed crystal structure of PDB (5frb) deposited structure. RMSD value ≤2 Å is generally acceptable docking protocol for model validation. Most active compounds from both series **3** and **5** and VT-1598, as a control inhibitor, were docked into the active site of lanosterol 14α-demethylase. The predicted binding affinity of analogues into the active site of the enzyme and the predicted polar contacts between them, as presented in Figure 2 showed that none of the synthesized pyrrole-carboxamides/carbohydrazides covalently interacted with the heme portion from the active site of sterol 14α-demethylase like classic antifungal azoles. In most of the antifungal drug candidates, triazole or tetrazole ring avails the metal-binding part on CYP51 to form coordinate bonds with the catalytic heme iron and exhibit off-target effects on human hepatic cytochrome P450 enzymes leading to liver toxicity. The docking study described herein showed that pyrrole-carbohydrazide and carboxamide (Figure 2) interact with the amino acids in the access channel to the active site of the sterol 14α-demethylase, and their interactions with either hydrogen bonds or π-π stacking with His374 of the enzyme are especially notable (Figure 2B1,C1). The His374 residue is highly conserved across the fungal kingdom and is fungus-specific. Perhaps this His374 interaction could contribute to retaining the high potency of these inhibitors against *A. fumigatus*.

The dimethylated pyrrole ring of all inhibitors is projected deeper into the CYP51 substrate binding cavity (Figure 2A1,A2) with the N-H orienting to form hydrogen bonding interactions with the hydroxyl of Tyr136 from the enzyme. The phenyl group and its substituents interact with His374, whereas, the dimethylated pyrrole ring of carboxamide analogues orient and form π-stacks to Tyr122 within the substrate access channel. Both the pyrrole-NH and carboxamide-NH form hydrogen bonds with Ser375 and the aromatic ring is aligned to π-stack with His374. The docking parameters (Glide scores) obtained for the best scored conformations show that the most active inhibitors display more favorable estimated binding free energy, −9.6 kcal/mol for compound **5h**, −9.23 for compound **5i**, −9.16 for compound **5j**, −7.82 kcal/mol for compound **3e** and −7.52 kcal/mol for compound **3k**, respectively, relative to VT-1598 (−12.08 kcal/mol).

**Figure 3:**
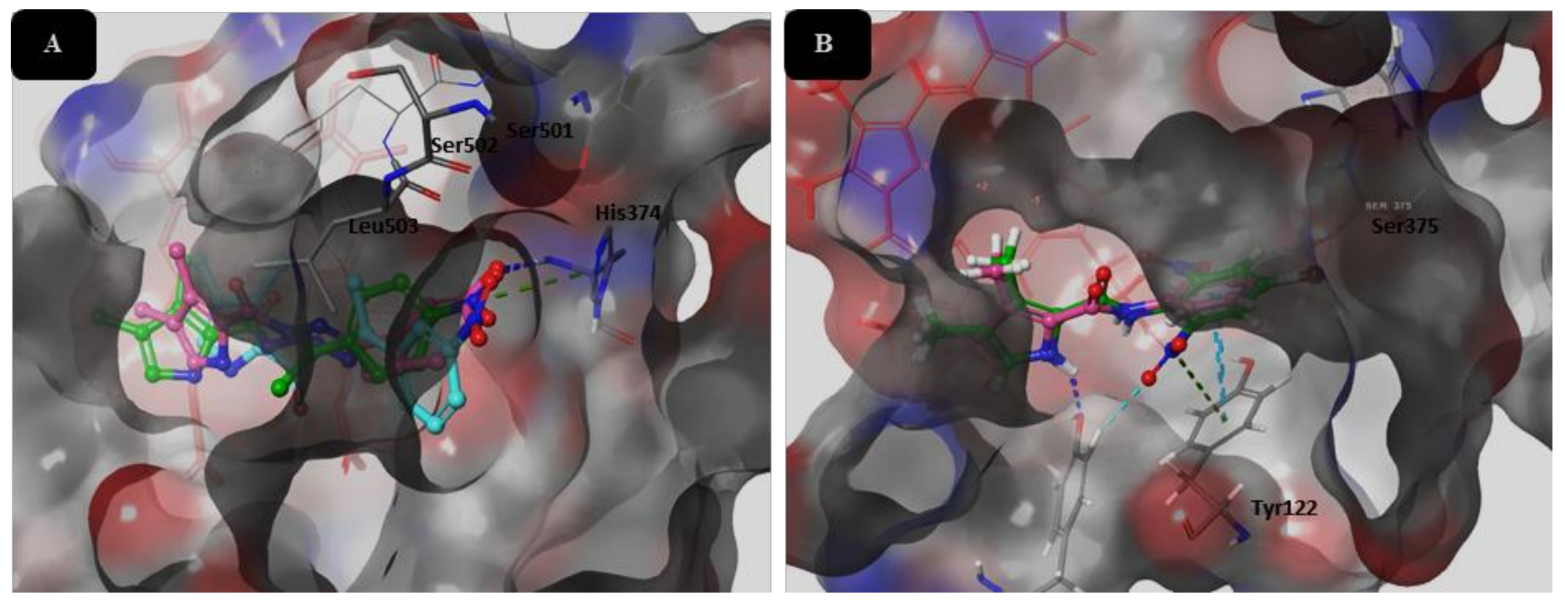
The representation of the orientation of aromatic substituents of most active compounds from (**A**) carbohydrazide compounds **5h** (blue), **5i** (green), **5j** (pink) and (**B**) carboxamide compounds **3h** (pink), **3k** (green)**,** in the active site of the CYP51B sterol 14α-demethylase of *A. fumigatus*.

Moreover, aromatic substituents (nitro or hydroxyl groups) from the most active carbohydrazide conformations were positioned toward the carbonyl groups of Leu503, Ser502, Ser203 and formed polar interactions with His374 (Figure 3A) whereas, aromatic ring-containing substituents (*p-*halo *or o-*dinitro) from the most active carboxamide conformations form π-stacks with Tyr122 and polar interactions with Ser375 (Figure 3B).

Based on the in vitro antifungal activity and molecular docking results, it was established that the synthesized compounds could inhibit the enzyme cytochrome P450 sterol 14α-demethylase of *A. fumigatus*. The docking score data further suggested that the novel analogues have good potential to inhibit the enzyme cytochrome P450 sterol 14α-demethylase.

## 3. Materials and Methods

### 3.1. Chemistry: General Information

All chemicals and reagents were obtained from commercial sources and were used as supplied, without further purification. The purification of synthesized compounds was performed when required, by recrystallization with an appropriate solvent system. Melting points were determined by the open glass capillary method and are uncorrected. The reaction progress and purity of the synthesized compounds were monitored by analytical thin layer chromatography (TLC) using precoated Silica Gel 60F 254 sheets (Merck, Darmstadt, Germany), a hexane-ethyl acetate 2:8 elution system and ultraviolet UV light (254 nm) for visualization. IR spectra were recorded using nujol oil with a Perkin Elmer (Waltham, MA, USA) FTIR spectrophotometer. Nuclear magnetic resonance (^1^H-NMR and ^13^C-NMR) spectra were recorded at room temperature on a Unity-300 spectrometer (300/75 MHz, Varian, Santa Clara, CA, USA) for compounds **3a**–**m** and an Avance NMR spectrometer (Bruker, Karlsruhe, Germany) operating at 400/100 MHz for compounds **5a**–**l**, using tetramethylsilane (TMS) as an internal standard (chemical shift in δ ppm). All NMRs were in accordance with the assigned structures. MS analyses were performed with a 4800 Plus MALDI TOF/TOF analyzer (Biosystems, Cheshire, UK) for compounds **3a**–**m** and a VG Platform spectrometer (Micromass, Beverly, MA, USA) for compounds **5a**–**l** in EZ-MS mode. The comprehensive spectral analysis including NMRs and HPLC data of active compounds is provided as Supplementary Figures S1–S15.

### 3.2. General Procedure for Synthesis of 3,4-Dimethyl-1H-pyrrole-2-carboxamides ***3a**–**m***

To a solution of 3,4-dimethyl-1*H*-pyrrole-2-carboxylic acid (1.0 eq.) in an appropriate volume of anhydrous acetonitrile (~5 mL per gram eq. of acid), were added EDC hydrochloride salt (1.2 eq.) and DMAP (1.2 eq.). The mixture was stirred for 15 min at room temperature under nitrogen environment. To this was added an appropriately substituted aniline **2a**–**m** (1.1 eq.) and the reaction mixture was further stirred for 8 h at room temperature. The reaction was quenched with water and desired compound was extracted in dichloromethane (×3). A combined organic layer was washed with 10% sodium bicarbonate (×3), water (×1), brine (×1) and then dried over anhydrous sodium sulphate. Dichloromethane was evaporated under reduced pressure to obtain the desired product.

*3,4-Dimethyl-N-phenyl-1H-pyrrole-2-carboxamide* (**3a**). Grey powder; Yield 87%; m.p. 188–190 °C; IR (KBr, cm^−1^) 3435 (N-H), 3281 (N-H), 1685 (C=O), 1459 (C=C); ^1^H-NMR (DMSO-*d_6_*, *δ* ppm) 2.05 (s, 3H, CH_3_), 2.40 (s, 3H, CH_3_), 6.69 (s, 1H, pyrrole C^5^-H), 7.09–7.14 (m, 1H, Ar-H), 7.31–7.38 (m, 2H, Ar-H), 7.53 (s, merged, pyrrole N^1^-H), 7.56–7.59 (m, 2H, Ar-H), 9.34 (bs, 1H, CONH); ^13^C-NMR (DMSO-*d_6_*, *δ* ppm) 9.9, 10.8, 119.5, 119.9, 120.3, 122.8, 123.9, 125.3, 128.9, 137.9, 159.9; MS *m/z* C_13_H_14_N_2_O 215.12 (M + H)^+^, found 215.30 (M + H)^+^. 

*3,4-Dimethyl-N-o-tolyl-1H-pyrrole-2-carboxamide* (**3b**). Grey powder; Yield: 85%; m.p. 192–194 °C; IR (KBr, cm^−1^) 3439 (NH), 3287 (NH), 1686 (C=O), 1462 (C=C); ^1^H-NMR (DMSO-*d_6_*, *δ* ppm) 2.06 (s, 3H, CH3), 2.34 (s, 3H, CH3), 2.39 (s, 3H, CH_3_), 6.72 (s, 1H, pyrrole C^5^-H), 7.04–7.09 (m, 1H, Ar-H), 7.20–7.25 (m, 3H, Ar-H), 8.09 (s, 1H, pyrrole N^1^-H), 9.28 (bs, 1H, CONH); ^13^C-NMR (DMSO-*d_6_*, *δ* ppm) 9.8, 10.4, 18.1, 119.9, 122.2, 124.4, 125.4, 125.4, 126.8, 127.7, 130.4, 136.2, 137.5, 159.9; MS *m/z* C_14_H_16_N_2_O 229.13 (M + H)^+^, found 229.30 (M + H)^+^.

*3,4-Dimethyl-N-p-tolyl-1H-pyrrole-2-carboxamide* (**3c**). Grey powder; Yield 87%; m.p. 212–214 °C; IR (KBr, cm^−1^) 3439 (NH), 3287 (NH), 1686 (C=O), 1462 (C=C); ^1^H-NMR (DMSO-*d_6_*, *δ* ppm) 2.04 (s, 3H, CH3), 2.32 (s, 3H, CH3), 2.40 (s, 3H, CH_3_), 6.66 (s, 1H, pyrrole C^5^-H), 7.12–7.14 (d, 2H, Ar-H), 7.44–7.46 (d, 2H, Ar-H), 7.49 (s, 1H, pyrrole N^1^-H), 9.36 (bs, 1H, CONH); ^13^C-NMR (DMSO-*d_6_*, *δ* ppm) 10.4, 10.8, 20.8, 119.5, 120.2, 122.9, 125.4, 129.5, 133.7, 135.4, 137.5, 160.0; MS *m/z* C_14_H_16_N_2_O 229.13 (M + H)^+^, found 229.20 (M + H)^+^.

*N-(2-Fluorophenyl)-3,4-dimethyl-1H-pyrrole-2-carboxamide* (**3d**). Grey powder; Yield 88%; m.p. 204–206 °C; IR (KBr, cm^−1^) 3446 (NH), 3279 (NH), 1685 (C=O), 1455 (C=C); ^1^H-NMR (DMSO-*d_6_*, *δ* ppm) 2.04 (s, 3H, CH_3_), 2.40 (s, 3H, CH_3_), 6.75 (s, 1H, pyrrole C^5^-H), 7.04–7.16 (m, 3H, Ar-H), 7.84 (bs, 1H, pyrrole N^1^-H), 8.44–8.49 (t, 1H, Ar-H), 9.21 (bs, 1H, CONH); ^13^C-NMR (DMSO-*d_6_*, *δ* ppm) 9.8, 10.4, 114.7, 119.8, 122.9, 123.6, 124.6, 125.4, 126.9, 137.5, 150.7, 151.6, 159.7; MS *m/z* C_13_H_13_FN_2_O 233.11 (M + H)^+^, found 233.10 (M + H)^+^.

*N-(4-Fluorophenyl)-3,4-dimethyl-1H-pyrrole-2-carboxamide* (**3e**). Grey powder; Yield 87%; m.p. 202–204 °C; IR (KBr, cm^−1^) 3434 (NH), 3305 (NH), 1684 (C=O), 1462 (C=C); ^1^H-NMR (DMSO-*d_6_*, *δ* ppm) 2.03 (s, 3H, CH_3_), 2.40 (s, 3H, CH_3_), 6.90 (s, 1H, pyrrole C^5^-H), 8.19–8.22 (dd, 4H, Ar-H), 9.10 & 9.11 (bs, 2H, merged, pyrrole N^1^-H & CONH); ^13^C-NMR (DMSO-*d_6_*, *δ* ppm): 9.7, 10.3, 115.7, 120.4, 121.9, 122.6, 125.3, 127.9, 137.5, 154.5, 159.9; MS *m/z* C_13_H_13_FN_2_O 233.11 (M + H)^+^, found 233.30 (M + H)^+^.

*N-(2-Chlorophenyl)-3,4-dimethyl-1H-pyrrole-2-carboxamide* (**3f**). Grey powder; Yield 86%; m.p. 198–200 °C; IR (KBr, cm^−1^) 3360 (NH), 3270 (NH), 1685 (C=O), 1460 (C=C); ^1^H-NMR (DMSO-*d_6_*, *δ* ppm) 2.07 (s, 3H, CH_3_), 2.39 (s, 3H, CH_3_), 6.76 (s, 1H, pyrrole C^5^-H), 7.02–7.07 (t, 1H, Ar-H), 7.31–7.33 (t, 1H, Ar-H), 7.42–7.43 (d, 1H, Ar-H), 8.15 (s, 1H, Ar-H), 8.56–8.60 (d, 1H, pyrrole N^1^-H), 9.20 (s, 1H, CONH); ^13^C-NMR (DMSO-*d_6_*, *δ* ppm) 9.7, 10.4, 119.7, 119.8, 119.9, 120.6, 121.2, 123.9, 125.4, 127.7, 128.9, 137.5, 151.5; MS *m/z* C_13_H_14_ClN_2_O 249.08 (M + H)^+^, found 249.10 (M + H)^+^.

*N-(4-Chlorophenyl)-3,4-dimethyl-1H-pyrrole-2-carboxamide* (**3g**). Pale Grey powder; Yield 87%; m.p. 198–200 °C; IR (KBr, cm^−1^) 3352 (NH), 3301 (NH), 1684 (C=O), 1460 (C=C); ^1^H-NMR (-*d_6_*, *δ* ppm) 2.03 (s, 3H, CH_3_), 2.37 (s, 3H, CH_3_), 6.73 (s, 1H, pyrrole C^5^-H), 7.29–7.32 (d, 2H, Ar-H), 7.46 (s, pyrrole N^1^-H), 7.52–7.55 (d, 2H, Ar-H), 9.13 (bs, 1H, CONH); ^13^C-NMR (DMSO-*d_6_*, *δ* ppm) 9.8, 10.0, 100.0, 119.8, 119.9, 120.2, 120.6, 121.2, 129.0, 134.8, 154.3; MS *m/z* C_13_H_14_ClN_2_O 249.08 (M + H)^+^, found 249.05 (M + H)^+^.

*N-(4-Bromophenyl)-3,4-dimethyl-1H-pyrrole-2-carboxamide* (**3h**). Grey powder; Yield 93%; m.p. 222–224 °C; IR (KBr, cm^−1^) 3360 (NH), 3302 (NH), 1685 (C=O), 1457 (C=C); ^1^H-NMR (DMSO-*d_6_*, *δ* ppm) 2.23 (s, 3H, CH_3_), 2.50 (s, 3H, CH_3_), 7.03 (s, 1H, pyrrole C^5^-H), 7.75–7.78 (d, 2H, Ar-H), 7.90–7.93 (d, 2H, Ar-H), 9.73 (bs, 1H, pyrrole N^1^-H), 11.30 (bs, 1H, CONH); ^13^C-NMR (DMSO-*d_6_*, *δ* ppm) 9.9, 10.8, 119.5, 119.9, 120.3, 122.8, 123.9, 125.3, 128.9, 137.9, 159.9; MS *m/z* C_13_H_14_BrN_2_O 293.03 (M + H)^+^, found 293.05 (M + H)^+^.

*3,4-Dimethyl-N-(2-nitrophenyl)-1H-pyrrole-2-carboxamide* (**3i**). Green powder; Yield 81%; m.p. 210–212 °C; IR (KBr, cm^−1^): 3360 (NH), 3290 (NH), 1684 (C=O), 1461 (C=C); ^1^H-NMR (DMSO-*d_6_*, *δ* ppm) 2.06 (s, 3H, CH_3_), 2.46 (s, 3H, CH_3_), 6.77 (s, 1H, pyrrole C^5^-H), 7.13–7.16 (t, 1H, Ar-H), 7.62–7.67 (t, 1H, ArH), 8.21–8.24 (d, 1H, Ar-H), 8.82–8.85 (d, 1H, ArH), 9.15 (s, 1H, pyrrole N^1^-H), 10.60 (s, 1H, CONH); ^13^C-NMR (DMSO-*d_6_*, *δ* ppm) 9.1, 9.8, 119.4, 120.5, 122.3, 124.8, 125.2, 127.8, 134.8, 135.1, 136.9, 150.9, 159.4; MS *m/z* C_13_H_13_N_3_O_3_ 260.10 (M + H)^+^, found 260.30 (M + H)^+^.

*3,4-Dimethyl-N-(3-nitrophenyl)-1H-pyrrole-2-carboxamide* (**3j**). Yellowish-green powder; Yield 83%; m.p. 112–114 °C; IR (KBr, cm^−1^) 3433 (NH), 3295 (NH), 1685 (C=O), 1460 (C=C); ^1^H-NMR (DMSO-*d_6_*, *δ* ppm) 2.01 (s, 3H, CH_3_), 2.18 (s, 3H, CH_3_), 6.93 (s, 1H, pyrrole C^5^-H), 6.95–6.96 (d, 1H, Ar-H), 7.23–7.29 (t, 1H, Ar-H), 7.47–7.57 (m, 2H, Ar-H), 8.50 (s, 1H, pyrrole N^1^-H), 9.35 (s, 1H, CONH); ^13^C-NMR (DMSO-*d_6_*, *δ* ppm) 9.7, 10.4, 108.9, 112.9, 118.4, 119.9, 120.6, 125.4, 129.8, 137.6, 147.5, 149.1, 151.5; MS *m/z* C_13_H_13_N_3_O_3_ 260.10 (M + H)^+^, found 260.20 (M + H)^+^.

*3,4-Dimethyl-N-(2,6-dinitrophenyl)-1H-pyrrole-2-carboxamide* (**3k**). Yellowish-green powder; Yield 82%; m.p. 180–182 °C; IR (KBr, cm^−1^) 3435 (NH), 3281 (NH), 1685 (C=O), 1459 (C=C); ^1^H-NMR (DMSO-*d_6_*, *δ* ppm) 2.05 (s, 3H, CH_3_), 2.36 (s, 3H, CH_3_), 6.70 (s, 1H, pyrrole C^5^-H), 7.00–7.06 (t, 1H, Ar-H), 7.46 (s, 1H, pyrrole N^1^-H), 7.50–7.55 (m, 2H, Ar-H), 9.21 (s, 1H, CONH); ^13^C-NMR (DMSO-*d_6_*, *δ* ppm) 10.4, 10.8, 20.8, 119.5, 120.2, 122.9, 125.4, 129.5, 133.7, 135.4, 137.5, 160.0; MS *m/z* C_13_H_12_N_4_O_5_ 305.09 (M + H)^+^, found 305.10 (M + H)^+^.

*N-(2-Methoxyphenyl)-3,4-dimethyl-1H-pyrrole-2-carboxamide* (**3l**). Grey powder; Yield 83%; m.p. 188–190 °C; IR (KBr, cm^−1^) 3430 (NH), 3273 (NH), 1684 (C=O), 1462 (C=C); ^1^H-NMR (DMSO-*d_6_*, *δ* ppm) 2.01 (s, 3H, CH_3_), 2.37 (s, 3H, CH_3_), 3.92 (s, 3H, OCH_3_), 6.71 (s, 1H, pyrrole C^5^-H), 6.88–6.91 (d, 1H, Ar-H), 6.97–6.99 (d, 1H, Ar-H), 7.0–7.03 (t, 1H, Ar-H), 8.33 (s, 1H, pyrrole N^1^-H), 8.48–8.51 (t, 1H, Ar-H), 9.49 (bs, 1H, CONH); ^13^C-NMR (DMSO-*d_6_*, *δ* ppm) 10.1, 10.5, 55.8, 109.8, 119.4, 119.9, 120.2, 121.2, 123.5, 125.3, 128.2, 137.5, 147.7, 159.7; MS *m/z* C_14_H_16_N_2_O_2_ 245.13 (M + H)^+^, found 245.10 (M + H)^+^.

*N-(4-Methoxyphenyl)-3,4-dimethyl-1H-pyrrole-2-carboxamide* (**3m**). Grey powder; Yield 85%; m.p. 192–194 °C; IR (KBr, cm^−1^) 3356 (NH), 3322 (NH), 1685 (C=O), 1463 (C=C); ^1^H-NMR (DMSO-*d_6_*, *δ* ppm) 2.05 (s, 3H, CH3), 2.36 (s, 3H, CH3), 3.80 (s, 3H, OCH3), 6.67 (s, 1H, pyrrole C^5^-H), 6.87–6.89 (d, 2H, Ar-H and 1H merged, pyrrole N^1^-H), 7.45–7.48 (d, 2H, Ar-H), 9.27 (bs, 1H, CONH); ^13^C-NMR (DMSO-*d_6_*, *δ* ppm) 10.1, 10.9, 55.5, 114.2, 120.2, 122.8, 125.4, 126.9, 130.9, 137.6, 156.4, 160.1; MS *m/z* C_14_H_16_N_2_O_2_ 245.13 (M + H)^+^, found 245.3 (M + H)^+^.

### 3.3. General Procedure for the Synthesis of 3,4-Dimethyl-1H-pyrrole-2-carbohydrazides ***5a**–**l***

To a solution of 3,4-dimethyl-1*H*-pyrrole-2-carbohydrazide (1 eq.) in a minimum volume of ethanol (~1.5 mL) was added a solution of a substituted acetophenone (**4a**–**l**, 1 eq.) in minimum ethanol (~1.5 mL). The reaction mixture was refluxed for 4–5 h, then cooled to room temperature and poured into ice cold water. The solid product suspended was collected by vacuum filtration and then dried at 70 °C. The desired product was recrystallized from ethanol and dried.

*(E)-3,4-Dimethyl-N’-(1-phenylethylidene)-1H-pyrrole-2-carbohydrazide* (**5a**). Off-white powder; Yield 88%; m.p. 196–198 °C; LCMS *R_t_* 2.75 min.; IR (KBr, cm^−1^) 3394 (NH), 3256 (NH), 1440 (C=O), 1527 (C=N), 1463 (C=C); ^1^H-NMR (DMSO-*d_6_*, *δ* ppm) 2.06 (s, 3H, CH_3_), 2.39 (s, 3H, CH_3_), 2.42 (s, 3H, CH_3_), 6.71 (s, 1H, pyrrole C^5^-H), 7.35–7.54 (m, 3H, ArH), 7.70 (d, 2H, ArH), 9.40 (s, 1H, pyrrole N^1^-H), 10.71 (s, 1H, N-NH); ^13^C-NMR (DMSO-*d_6_*, *δ* ppm) 9.9, 11.0, 13.6, 119.9, 120.1, 126.3, 126.3, 128.7, 129.3, 131.2, 134.8, 138.7, 141.5, 154.6; MS *m/z* C_15_H_18_N_3_O 256.14 (M + H)^+^, found 256.10 (M + H)^+^.

*(E)-3,4-Dimethyl-N’-(1-p-tolylethylidene)-1H-pyrrole-2-carbohydrazide* (**5b**). White powder; Yield 92%; m.p. 218–220 °C; LCMS *R_t_* 2.88 min.; IR (KBr, cm^−1^) 3388 (NH), 3259 (NH), 1639 (C=O), 1526 (C=N), 1463 (C=C); ^1^H-NMR (DMSO-*d_6_*, *δ* ppm) 2.06 (s, 3H, CH_3_), 2.35 (s, 3H, CH_3_), 2.40 (s, 3H, CH_3_), 2.42 (s, 3H, CH_3_), 6.70 (d, 1H, pyrrole C^5^-H), 7.23–7.25 (dd, 2H, Ar-H), 7.58–7.60 (d, 2H, Ar-H), 9.17 (s, 1H, pyrrole N^1^-H), 10.76 (s, 1H, N-NH); ^13^C-NMR (DMSO-*d_6_*, *δ* ppm) 9.9, 11.0, 13.4, 21.3, 120.0, 121.9, 126.0, 129.4, 133.6, 139.5, 142.7, 151.8, 156.4; MS *m/z* C_16_H_19_N_3_O 270.16 (M + H)^+^, found 270.20 (M + H)^+^.

*(E)-N’-(1-(4-Aminophenyl)ethylidene)-3,4-dimethyl-1H-pyrrole-2-carbohydrazide* (**5c**). Light-yellow powder; Yield 92%; m.p. 210–212 °C; LCMS *R_t_* 2.34 min.; IR (KBr, cm^−1^) 3448 (NH), 3398 (NH), 3319 (NH), 3247 (NH), 1638 (C=O), 1511 (C=N), 1463 (C=C); ^1^H-NMR (DMSO-*d_6_*, *δ* ppm) 1.96 (s, 3H, CH_3_), 2.22 (d, 6H, 2xCH_3_), 5.44 (s, 2H, NH_2_), 6.56–6.58 (d, 2H, Ar-H), 6.71 (s, 1H, pyrrole C^5^-H), 7.51–7.53 (d, 2H, Ar-H), 9.58 (s, 1H, pyrrole N^1^-H), 11.06 (s, 1H, N-NH); ^13^C-NMR (DMSO-*d_6_*, *δ* ppm) 10.3, 11.0, 14.0, 113.7, 118.8, 119.5, 126.0, 127.4, 127.9, 129.7, 144.2, 150.5, 157.18; MS *m/z* C_15_H_18_N_4_O 271.16 (M + H)^+^, found 271.20 (M + H)^+^.

*(Z)-N’-(1-(4-Fluorophenyl)ethylidene)-3,4-dimethyl-1H-pyrrole-2-carbohydrazide* (**5d**). Off white powder; Yield 86%; m.p. 214–216 °C; LCMS *R_t_* 2.77 min.; IR (KBr, cm^−1^) 3395 (NH), 3266 (NH), 1642 (C=O), 1500 (C=N), 1463 (C=C); ^1^H-NMR (DMSO-*d_6_*, *δ* ppm) 1.96 (s, 3H, CH_3_), 2.23 (s, 3H, CH_3_), 2.32 (s, 3H, CH_3_), 6.73 (s, 1H, pyrrole C^5^-H), 7.23–7.27 (t, 2H, Ar-H), 7.83–7.87 (m, 2H, Ar-H), 9.82 (s, 1H, pyrrole N^1^-H), 11.12 (s, 1H, N-NH); ^13^C-NMR (DMSO-*d_6_*, *δ* ppm) 10.3, 11.0, 14.4, 115.6, 115.8, 118.976, 119.5, 120.0, 128.8, 128.9, 132.3, 135.4, 164.9, 169.0; MS *m/z* C_15_H_17_FN_3_O 274.14 (M + H)^+^, found 274.50 (M + H)^+^.

*(E)-N’-(1-(4-Chlorophenyl)ethylidene)-3,4-dimethyl-1H-pyrrole-2-carbohydrazide* (**5e**). White powder; Yield 87%; m.p. 226–228 °C; LCMS *R_t_* 2.88 min.; IR (KBr, cm^−1^) 3382 (NH), 3259 (NH), 1639 (C=O), 1522 (C=N), 1463 (C=C); ^1^H-NMR (DMSO-*d_6_*, *δ* ppm) 1.96 (s, 3H, CH_3_), 2.23 (s, 3H, CH_3_), 2.32 (s, 3H, CH_3_), 6.74 (s, 1H, pyrrole C^5^-H), 7.47–7.49 (d, 2H, Ar-H), 7.81–7.83 (d, 2H, Ar-H), 9.86 (s, 1H, pyrrole N^1^-H), 11.13 (s, 1H, N-NH); ^13^C-NMR (DMSO-*d_6_*, *δ* ppm) 10.3, 11.0, 14.2, 119, 120.1, 121.6, 128.4, 128.8, 134.7, 149.7, 151.4, 168.2; MS *m/z* C_15_H_17_ClN_3_O 290.11 (M + H)^+^, found 290.20 (M + H)^+^.

*(E)-N’-(1-(2,6-Dichlorophenyl)ethylidene)-3,4-dimethyl-1H-pyrrole-2-carbohydrazide* (**5f**). Off white powder; Yield 79%; m.p. 200–202 °C; LCMS *R_t_* 3.02 min.; IR (KBr, cm^−1^) 3395 (NH), 3267 (NH), 1640 (C=O), 1505 (C=N), 1462 (C=C); ^1^H-NMR (DMSO-*d_6_*, *δ* ppm) 1.95 (s, 3H, CH_3_), 2.22 (s, 3H, CH_3_), 2.30 (s, 3H, CH_3_), 6.73 (s, 1H, pyrrole C^5^-H), 7.48 (s, 2H, Ar-H), 7.69 (s, 1H, Ar-H), 9.90 (s, 1H, pyrrole N^1^-H), 11.10 (s, 1H, N-NH); ^13^C-NMR (DMSO-*d_6_*, *δ* ppm) 10.3, 11.0, 18.3, 127.928, 127.959, 127.980, 129.633, 129.643, 132.2, 132.8, 133.2; MS *m/z* C_15_H_16_Cl_2_N_3_O 324.07 (M + H)^+^, found 324.10 (M + H)^+^.

*(E)-N’-(1-(4-Bromophenyl)ethylidene)-3,4-dimethyl-1H-pyrrole-2-carbohydrazide* (**5g**). White powder; Yield 92%; m.p. 218–220 °C; LCMS *R_t_* 2.93 min.; IR (KBr, cm^−1^) 3380 (NH), 3260 (NH), 1638 (C=O), 1520 (C=N), 1462 (C=C); ^1^H-NMR (DMSO-*d_6_*, *δ* ppm) 1.96 (s, 3H, CH_3_), 2.23 (s, 3H, CH_3_), 2.31 (s, 3H, CH_3_), 6.74 (s, 1H, pyrrole C^5^-H), 7.60–7.63 (d, 2H, Ar-H), 7.74–7.77 (d, 2H, Ar-H), 9.86 (s, 1H, Pyrrole N^1^-H), 11.14 (s, 1H, N-NH); ^13^C-NMR (DMSO-*d_6_*, *δ* ppm) 10.3, 11.0, 14.1, 119.0, 120.1, 121.6, 122.9, 128.7, 131.7, 138.1, 144.7, 149.7, 159.1; MS *m/z* C_15_H_17_BrN_3_O 334.06 (M + H)^+^, found 334.00 (M + H)^+^.

*(E)-3,4-Dimethyl-N’-(1-(3-nitrophenyl)ethylidene)-1H-pyrrole-2-carbohydrazide* (**5h**). Yellow powder; Yield 73%; m.p. 238–240 °C; LCMS *R_t_* 2.73 min.; IR (KBr, cm^−1^) 3420 (NH), 3257 (NH), 1631 (C=O), 1525 (C=N), 1463 (C=C); ^1^H-NMR (DMSO-*d_6_*, *δ* ppm) 1.97 (s, 3H, CH_3_), 2.24 (s, 3H, CH_3_), 2.40 (s, 3H, CH_3_), 6.76 (s, 1H, pyrrole C^5^-H), 7.70–7.74 (t, 1H, Ar-H), 8.21–8.24 (m, 2H, Ar-H), 8.60 (s, 1H, Ar-H), 10.01 (s, 1H, pyrrole N^1^-H), 11.19 (s, 1H, N-NH); ^13^C-NMR (DMSO-*d_6_*, *δ* ppm) 10.3, 11.0, 14.2, 119.1, 120.2, 120.9, 121.4, 123.8, 130.5, 132.9, 140.6, 148.5; MS *m/z* C_15_H_17_N_4_O_3_ 301.13 (M + H)^+^, found 301.10 (M + H)^+^.

*(Z)-3,4-Dimethyl-N’-(1-(4-nitrophenyl)ethylidene)-1H-pyrrole-2-carbohydrazide* (**5i**). Yellow powder; Yield 76%; m.p. 258–260 °C; LCMS *R_t_* 2.77 min.; IR (KBr, cm^−1^) 3409 (NH), 3261 (NH), 1625 (C=O), 1510 (C=N), 1462 (C=C); ^1^H-NMR (DMSO-*d_6_*, *δ* ppm) 1.97 (s, 3H, CH_3_), 2.24 (s, 3H, CH_3_), 2.39 (s, 3H, CH_3_), 6.77 (s, 1H, pyrrole C^5^-H), 8.04–8.064 (d, 2H, Ar-H), 8.26–8.28 (d, 2H, Ar-H), 10.04 (s, 1H, pyrrole N^1^-H), 11.2 (s, 1H, N-NH); ^13^C-NMR (DMSO-*d_6_*, *δ* ppm) 10.3, 11.0, 14.2, 119.1, 120.4, 121.4, 124.0, 124.5, 127.7, 145.0, 147.8, 148.1, 156.3; MS *m/z* C_15_H_17_N_4_O_3_ 301.13 (M + H)^+^, found 301.10 (M + H)^+^.

*(E)-N’-(1-(3-Hydroxyphenyl)ethylidene)-3,4-dimethyl-1H-pyrrole-2-carbohydrazide* (**5j**). Off white powder; Yield 83%; m.p. 238–240 °C; LCMS *R_t_* 2.57 min.; IR (KBr, cm^−1^) 3451 (NH), 3399 (NH), 3267 (OH), 1639 (C=O), 1521 (C=N), 1457 (C=C); ^1^H-NMR (DMSO-*d_6_*, *δ* ppm) 1.96 (s, 3H, CH_3_), 2.24 (s, 3H, CH_3_), 2.28 (s, 3H, CH_3_), 6.74 (s, 1H, pyrrole C^5^-H), 6.79–6.81 (m, 1H, Ar-H), 7.20–7.22 (m, 2H, Ar-H), 7.26 (s, 1H, Ar-H), 9.51 (s, 1H, OH), 9.77 (s, 1H, pyrrole N^1^-H), 11.12 (s, 1H, N-NH); ^13^C-NMR (DMSO-*d_6_*, *δ* ppm) 10.3, 11.0, 14.4, 113.2, 116.7, 117.7, 118.9, 119.9, 121.8, 123.8, 129.8, 140.2, 149.9, 157.8; MS *m/z* C_15_H_18_N_3_O_2_ 272.14 (M + H)^+^, found 272.00 (M + H)^+^.

*(E)-N’-(1-(4-Methoxyphenyl)ethylidene)-3,4-dimethyl-1H-pyrrole-2-carbohydrazide* (**5k**). Off white powder; Yield 87%; m.p. 188–190 °C; LCMS *R_t_* 2.75 min.; IR (KBr, cm^−1^) 3383 (NH), 3265 (NH), 1639 (C=O), 1525 (C=N), 1462 (C=C); ^1^H-NMR (DMSO-*d_6_*, *δ* ppm) 1.96 (s, 3H, CH_3_), 2.24 (s, 3H, CH_3_), 2.30 (s, 3H, CH_3_), 3.80 (s, 3H, OCH_3_), 6.73 (s, 1H, pyrrole C^5^-H), 6.97–6.99 (d, 2H, Ar-H), 7.75–7.77 (d, 2H, Ar-H), 9.73 (s, 1H, pyrrole N^1^-H), 11.10 (s, 1H, N-NH); ^13^C-NMR (DMSO-*d_6_*, *δ* ppm) 10.3, 11.0, 14.2, 55.7, 114.2, 118.9, 119.8, 121.8, 128.1, 131.3, 137.5, 155.0, 160.5; MS *m/z* C_16_H_20_N_3_O_2_ 286.16 (M + H)^+^, found 286.10 (M + H)^+^.

*(Z)-N’-(1-(3,4-Dimethoxyphenyl)ethylidene)-3,4-dimethyl-1H-pyrrole-2-carbohydrazide* (**5l**). White powder; Yield 97%; m.p. 190–192 °C; LCMS *R_t_* 2.66 min.; IR (KBr, cm^−1^) 3350 (NH), 3261 (NH), 1628 (C=O), 1516 (C=N), 1463 (C=C); ^1^H-NMR (DMSO-*d_6_*, *δ* ppm) 1.96 (s, 3H, CH_3_), 2.23 (s, 3H, CH_3_), 2.30 (s, 3H, CH_3_), 3.80 (s, 6H, 2 OCH_3_), 6.73 (s, 1H, pyrrole C^5^-H), 6.98–7.00 (d, 1H, Ar-H), 7.31–7.33 (d, 1H, Ar-H), 7.43 (s, 1H, Ar-H), 9.77 (s, 1H, pyrrole N^1^-H), 11.08 (s, 1H, N-NH); ^13^C-NMR (DMSO-*d_6_*, *δ* ppm) 10.3, 11.0, 14.3, 56.0, 104.4, 111.6, 118.9, 119.7, 120.0, 121.7, 127.3, 132.1, 146.6, 149.0, 150.4, 154.1; MS *m/z* C_17_H_22_N_3_O_3_ 316.17 (M + H)^+^, found 316.30 (M + H)^+^.

### 3.4. Bioassays

In vitro antimicrobial activities of all the derivatives **3a**–**m** and **5a**–**l** were determined against different microorganisms by micro broth dilution assay as described earlier [39,40]. The microbial strains *Escherichia coli* NCIM 2065, *Pseudomonas aeruginosa* NCIM 5031, *Salmonella typhi* NCIM 2501, *Klebsiella pneumoniae* NCIM 2957, *Bacillus subtilis* NCIM 2699, *Aspergillus flavus* NCIM 549, *Aspergillus fumigatus* NCIM 902, *Aspergillus niger* NCIM 620 were obtained from the National Chemical Laboratory, Pune, India. The bacteria were maintained on nutrient broth (NB) and fungal strains were maintained on Sabouraud dextrose broth at 37 °C. Minimum Inhibitory Concentration (MIC) determination was carried out using the micro broth dilution method as per NCCLS guidelines [41,42].

#### 3.4.1. In Vitro Antibacterial Assay

The antibacterial activities were assayed against five bacterial strains: *Pseudomonas aeruginosa*, *Escherichia coli*, *Klebsiella pneumoniae*, *Salmonella typhi* and *Bacillus subtilis*. The bacterial strains used as inoculums were grown at 37 °C to get the optical density of 0.6 at 600 nm. Colony forming units (CFU) were counted by using serial plate dilution method and bacterial counts were adjusted to 1 × 10^5^ to 1 × 10^6^ CFU/mL for susceptibility testing. The microdilution broth method with Mueller-Hinton broth (Hi-Media Laboratories, Mumbai, India) adjusted to pH 7.4 (±0.2) was used. The tested compounds were dissolved in DMSO to final concentrations ranging from 10.0 to 0.019 mg/mL. Tetracycline was used as the reference drug. The test was performed in 96-wells culture plate (Hi-media). The compounds were dissolved in DMSO to prepare 10 serial dilutions viz. 10.0, 5.0, 2.5, 1.25, 0.625, 0.312, 0.156, 0.078, 0.039 and 0.019 mg/mL in the wells by twofold dilution method. The negative control was prepared using dimethyl sulphoxide (DMSO), and concentrations up to 0.000156 mg/mL of tetracycline used as positive control for bacteria. The 96 wells plates were incubated for 24 h at 37 °C. The lowest concentration of each compound which inhibited any visual growth was considered the MIC of that respective compound. All such experiments were repeated thrice and the results (MIC values as mg/mL) for compounds **3a**–**m** for respective bacterial species have been represented in tabular form in Table 1.

#### 3.4.2. In Vitro Antifungal Assay

The antifungal properties were evaluated against three *Aspergillus* strains (*Aspergillus niger, Aspergillus fumigatus* and *Aspergillus flavus).* The microbroth dilution method buffered to pH 7.0 with 0.165 mol of 3-morpholino-propane-1-sulphonic acid (Sigma-Aldrich, Schnelldorf, Germany). DMSO served as a diluent for all the compounds. The fungal inoculums were prepared from 10 days old culture grown on potato dextrose agar medium. The Petri dishes were flooded with 8 to 10 mL of distilled water and the conidia were scraped using the sterile spatula. The spore density of each fungus was adjusted with a spectrophotometer (A595 nm) to obtain a final concentration approximately 105 spores/mL. Amphotericin-B (concentration range of 1.0 mg/mL to 0.00015 mg/L) and fluconazole (concentration range of 1.0 mg/mL to 0.00061 mg/L) were involved as the reference drugs. The compounds were dissolved in DMSO to prepare 10 serial dilutions viz. 10.0, 5.0, 2.5, 1.25, 0.625, 0.312, 0.156, 0.078, 0.039, and 0.019 mg/mL in the wells by double dilution method. The 96 well plates were incubated for 48 h at 37 °C. The MIC values were assayed as inhibition of growth compared to the control. The results were analyzed visually. The MIC values were determined after 48 h of incubation in the dark at 37 °C (Table 2).

### 3.5. Molecular Docking

A molecular docking study was carried out using Glide (Schrodinger LLC, New York, NY, USA), [33,43] to investigate the possible interactions with cytochrome P450 14 α-sterol demethylase (CYP51B) from *Aspergillus fumigatus* and to predict and compare the binding interactions of our most active carboxamides **3e**, **3h**, **3k** and carbohydrazides **5h**, **5i**, **5j** with co-crystallized ligand VT-1598 [38]. The crystallographic structure of the complex between the X-ray structure of the enzyme and co-crystallized VT-1598 (PDB:5FRB), was employed for the docking study. A dataset of 3D pharmacophore conformers of lower energy of novel analogues **3e**, **3h**, **3k** and **5h**–**j** was prepared using the Ligprep (Schrodinger LLC) module. Further, protein preparation mode was used to minimized enzyme bound with VT-1598, a drug known to inhibit cytochrome P450 sterol 14α-demethylase, was taken as the positive control. Using Glide XP (extra precision)-docking mode, the best conformer pose for each docked compound were obtained. For each compound, Glide Docked searched for the five best conformers and binding poses with high Glide scores were selected to verify their binding pattern by visual analysis.

## 4. Conclusions

A series of aryl substituted 3,4-dimethyl-1*H*-pyrrole-2-carboxamides **3a**–**m** and 3,4-dimethyl-1*H*-pyrrole-2-carbohydrazides **5a**–**l** were synthesized using a one-pot method with a broad range of substrates scope under mild reaction conditions. The compounds were evaluated for their antibacterial and antifungal activities. The chemical structures of the newly synthesized molecules were characterized by modern spectroscopic methods (IR, ^1^H-NMR, ^13^C-NMR, MS). The general observations herein revealed that both series of active compounds exhibit better antifungal profile against *A. fumigatus* than antibacterial activity. However, the antibacterial profile was found to be improved with increasing electron withdrawing groups on the aromatic ring. Among all, compounds **5h**, **5i** and **5j** (from the carbohydrazide series) were the most potent against *A. fumigatus*, with MIC values of 0.039 mg/mL, around two-fold less potent than the reference fluconazole (MIC 0.0195 mg/mL). Albeit slightly potent an identical pattern was observed in compounds **3e**, **3h** and **3k** (carboxamide series) against *A. fumigatus*, with MIC values of 0.156 mg/mL. Compound **3k**, with *o-*dinitro aromatic substitution showed decently potent antibacterial activity against *E. coli* (MIC 0.078 mg/mL) whereas compound **5f**, with *o-*dichloro aromatic substitution exhibited decent but broad spectrum antibacterial activity against *P. aeruginosa, K. pneumoniae* (MICs 0.078 mg/mL) and *S. typhi* (MIC 0.039 mg/mL). Some other compounds among the synthesized derivatives were found to be moderately antibacterial, with the MIC values in the range of 0.312–0.625 mg/mL.

Furthermore, the most potent antifungal compounds **3j**–**k** and **5h**–**j** when docked inside the active site of sterol 14α-demethylase (CYP51B) showed hydrogen bonding and other bonding interactions along the access channel in the active site of sterol 14α-demethylase, where their π-stacking and/or H-bonding (Figure 2) alignment was notable for fungus-specific His374. None of the docked compounds showed any interactions with the catalytic heme iron part responsible for off-target liver toxicity-related side effects in most commercial antifungal compounds. In summary, these newly developed truncated pharmacophores show facility for structural permutations to develop a combinatorial approach for discovering broad spectrum antifungal agents.

## Figures and Tables

**Table:** 

Compounds (Yield)				R^1^	Compounds (Yield)				R^1^
2a	3a (87%)	4a	5a (88%)	H	2h	3h (93%)	4g	5g (92%)	4-Br
2b	3b (85%)	-	-	2-Me	2i	3i (81%)	-	-	2-NO_2_
2c	3c (87%)	4b	5b (92%)	4-Me	2j	3j (83%)	4h	5h (73%)	3-NO_2_
-	-	4c	5c (92%)	4-NH_2_	-	-	4i	5i (76%)	4-NO_2_
2d	3d (88%)	-	-	2-F	2k	3k (82%)	-	-	2,6-di-NO_2_
2e	3e (87%)	4d	5d (86%)	4-F	-	-	4j	5j (83%)	3-OH
2f	3f (86%)	-	-	2-Cl	2l	3l (83%)	-	-	2-OMe
2g	3g (87%)	4e	5e (87%)	4-Cl	2m	3m (85%)	4k	5k (87%)	4-OMe
-	-	4f	5f (79%)	2,6-di-Cl	-	-	4l	5l (97%)	3,4-di-OMe

**Table 1:** Antibacterial profile of dimethyl-1*H*-pyrrole-2-caboxamide and carbohydrazide analogues.

MIC (mg/mL)											
Comp.	PA	EC	KP	ST	BS	Comp.	PA	EC	KP	ST	BS
**3a**	0.625	0.625	1.25	1.25	1.25	**5a**	1.25	2.5	1.25	0.312	1.25
**3b**	1.25	0.625	1.25	1.25	1.25	**5b**	2.5	2.5	2.5	0.625	1.25
**3c**	1.25	0.625	1.25	1.25	1.25	**5c**	0.312	0.312	1.25	0.312	0.625
**3d**	0.625	0.625	1.25	1.25	0.625	**5d**	2.5	1.25	0.625	2.5	2.5
**3e**	0.625	0.625	1.25	1.25	1.25	**5e**	2.5	2.5	1.25	0.312	1.25
**3f**	1.25	0.625	1.25	1.25	1.25	**5f**	0.078	5.0	0.078	0.039	1.25
**3g**	0.625	0.625	1.25	0.625	1.25	**5g**	1.25	2.5	2.5	0.156	1.25
**3h**	1.25	0.625	1.25	1.25	1.25	**5h**	1.25	2.5	1.25	1.25	1.25
**3i**	0.625	0.625	0.625	1.25	1.25	**5i**	0.312	0.312	0.312	0.312	1.25
**3j**	0.312	0.625	0.625	0.625	0.625	**5j**	1.25	1.25	1.25	1.25	1.25
**3k**	0.312	0.078	0.625	0.625	0.625	**5k**	2.5	1.25	0.625	2.5	2.5
**3l**	1.25	0.625	1.25	1.25	1.25	**5l**	2.5	1.25	2.5	2.5	2.5
**3m**	1.25	0.625	1.25	1.25	1.25	**TC**	0.00125	0.01	0.000312	0.01	0.00125

PA: *Pseudomonas aeruginosa*; EC: *Escherichia coli*; KP: *Klebsiella pneumoniae*; ST: *Salmonella typhi*; BS: *Bacillus subtilis*; TC: Tetracycline.

**Table 2:** Antifungal profile of dimethyl-1H-pyrrole-2-caboxamide and carbohydrazide analogues.

MIC (mg/mL)							
Comp	*Aspergillus niger*	*Aspergillus fumigatus*	*Aspergillus flavus*	Comp.	*Aspergillus niger*	*Aspergillus fumigatus*	*Aspergillus flavus*
**3a**	0.625	0.625	0.312	**5a**	0.625	1.25	1.25
**3b**	0.625	0.625	0.312	**5b**	0.078	0.625	0.156
**3c**	0.625	0.312	0.312	**5c**	0.312	0.156	0.156
**3d**	0.625	0.625	0.625	**5d**	1.25	0.156	0.625
**3e**	0.312	0.156	0.312	**5e**	0.078	1.25	1.25
**3f**	0.312	0.312	0.312	**5f**	1.25	0.156	0.625
**3g**	0.625	0.625	0.625	**5g**	0.078	2.5	1.25
**3h**	0.625	0.156	0.625	**5h**	1.25	0.039	0.625
**3i**	0.625	0.625	0.625	**5i**	1.25	0.039	1.25
**3j**	0.312	0.312	0.156	**5j**	0.312	0.039	0.312
**3k**	0.312	0.156	0.312	**5k**	1.25	0.156	0.625
**3l**	0.625	0.625	0.156	**5l**	1.25	0.312	1.25
**3m**	0.625	0.625	0.312	-	-	-	-
AMP *	0.00125	0.000156	0.000156	FCZ *	0.00061	0.0195	0.0049

* AMP amphotericin B; FCZ fluconazole.

## Supplementary Materials

The following are available online.
