# Supplementary material for: Facile Syntheses and Molecular-Docking of Novel Substituted 3,4-Dimethyl-1H-pyrrole-2-carboxamide/carbohydrazide Analogues with Antimicrobial and Antifungal Properties
Source: Molecules. 2018 Apr 11;23(4):875. doi: 10.3390/molecules23040875 (PMC6017109; doi:10.3390/molecules23040875)

Supplementary:

Figure S1 HNMR Spectrum of compound 5h in DMSO
Figure S2 C13-NMR Spectrum of compound 5h in DMSO
Figure S3 LCMS Spectrum with retention time (min) of compound 5h
Figure S4 MS of compound 5h
Figure S5 HNMR Spectrum of compound 5i in DMSO
Figure S6 C13-NMR Spectrum of compound 5i in DMSO
Figure S7 LCMS Spectrum with retention time (min) of compound 5i
Figure S8 MS of compound 5i
Figure S9 HNMR Spectrum of compound 5j in DMSO
Figure S10 C13-NMR Spectrum of compound 5j in DMSO
Figure S11 LCMS Spectrum with retention time (min) of compound 5j
Figure S12 MS of compound 5j
Figure S13 HNMR Spectrum of compound 3e in DMSO
Figure S14 HNMR Spectrum of compound 3h in DMSO
Figure S15 HNMR Spectrum of compound 3k in DMSO

Figure S1 HNMR Spectrum of compound 5h in DMSO solvent

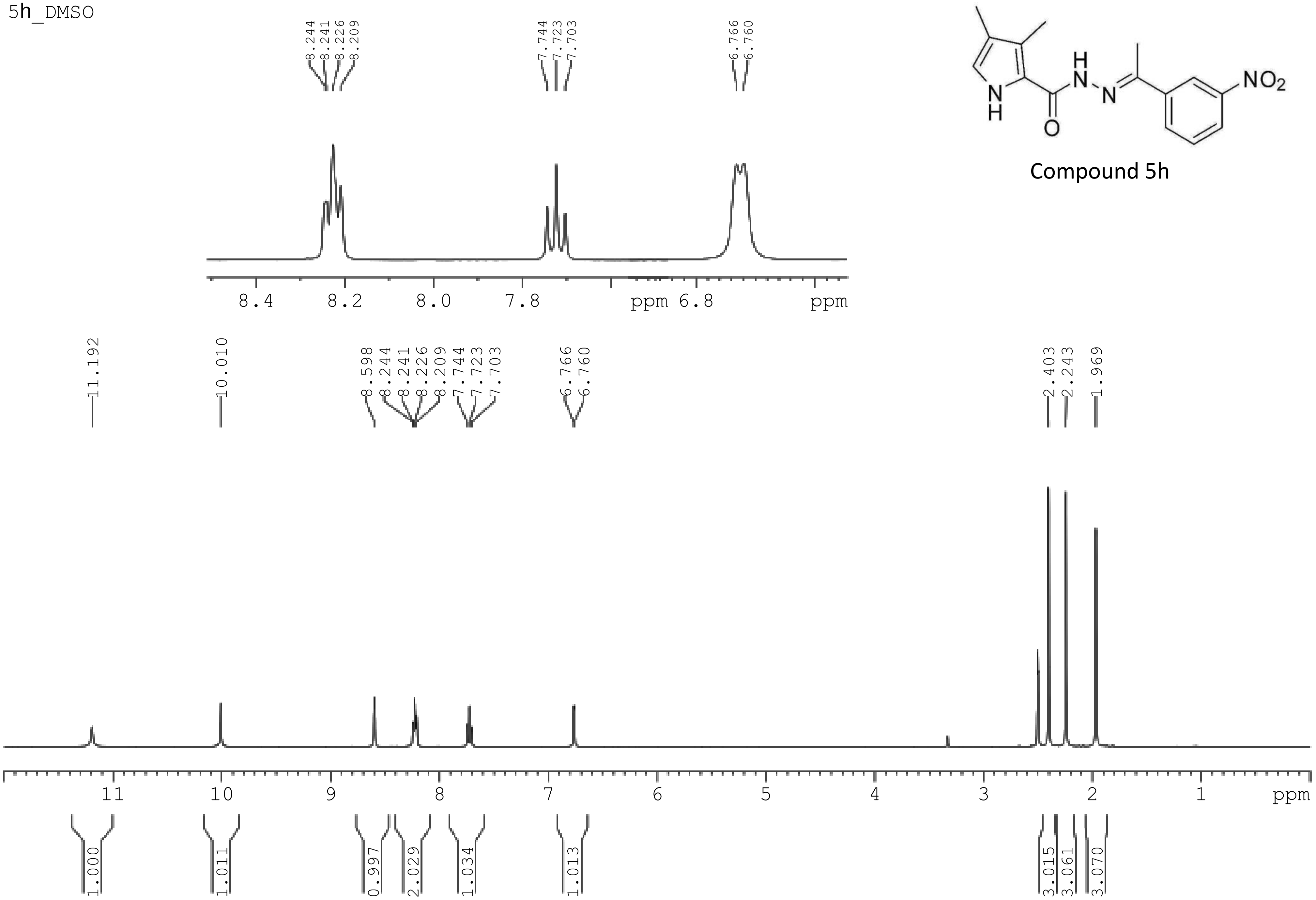

Compound 5h

Figure S2 C13-NMR Spectrum of compound 5h in DMSO

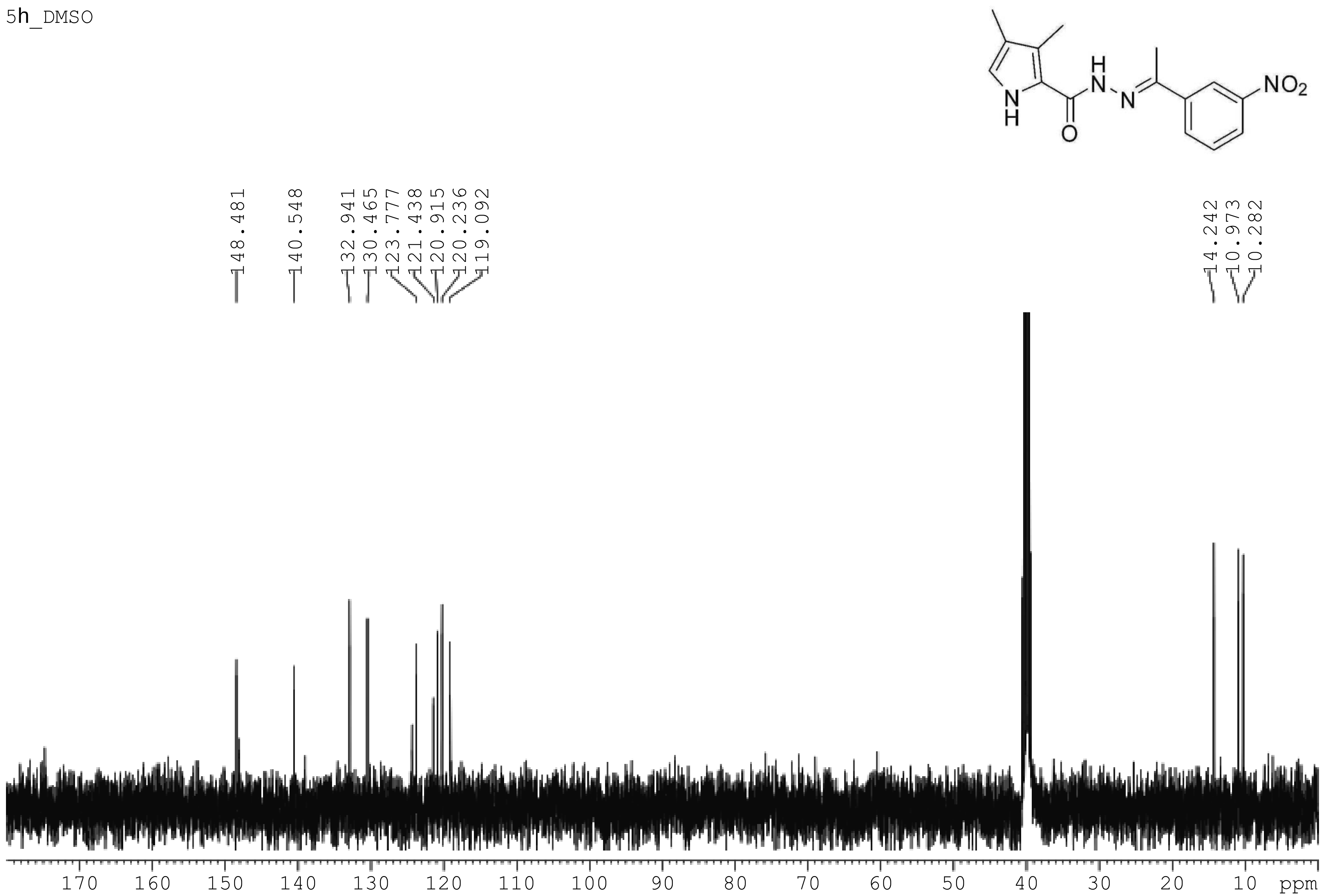

Figure S3 LCMS Spectrum with retention time (min) of compound 5h

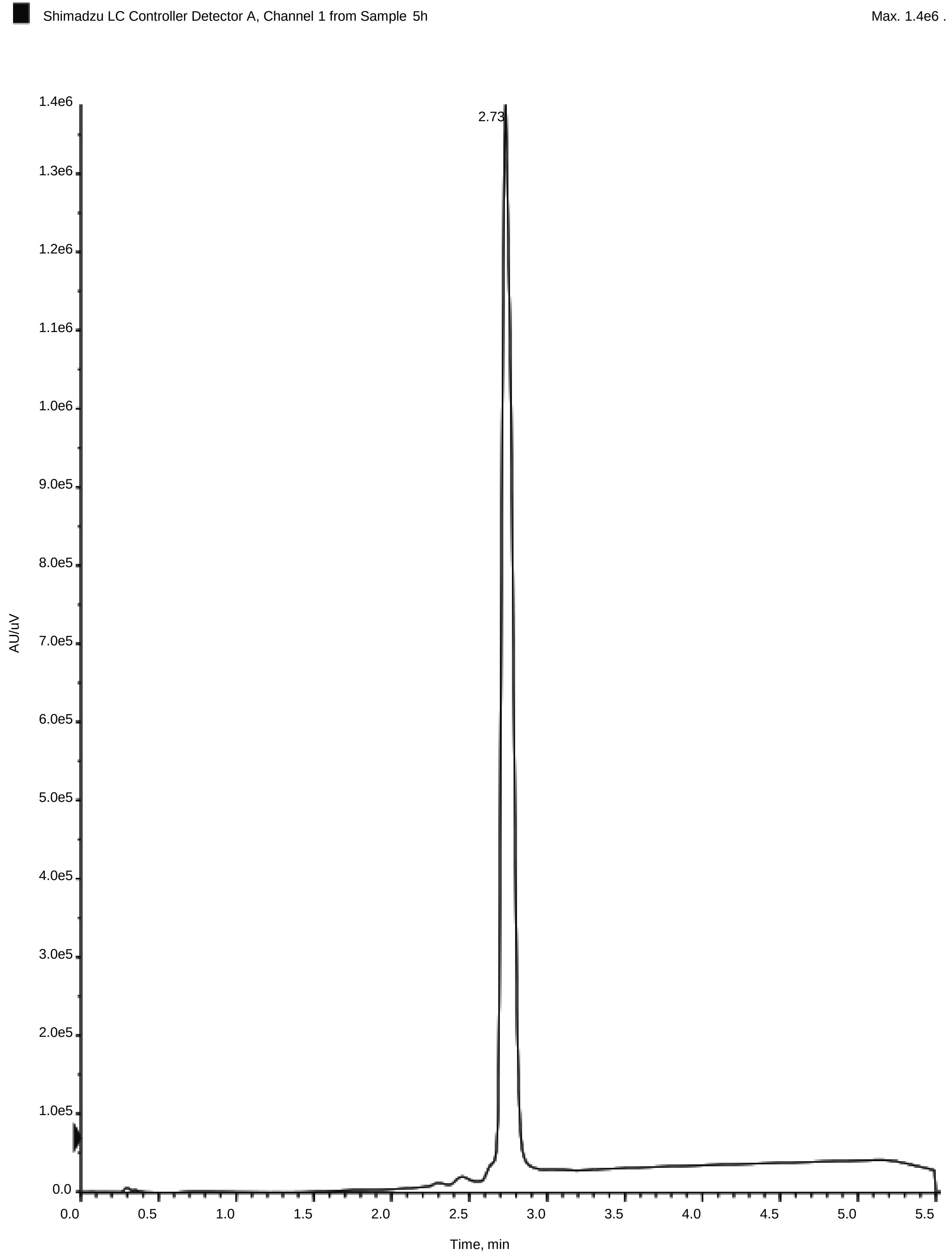

Figure S4 MS of compound 5h

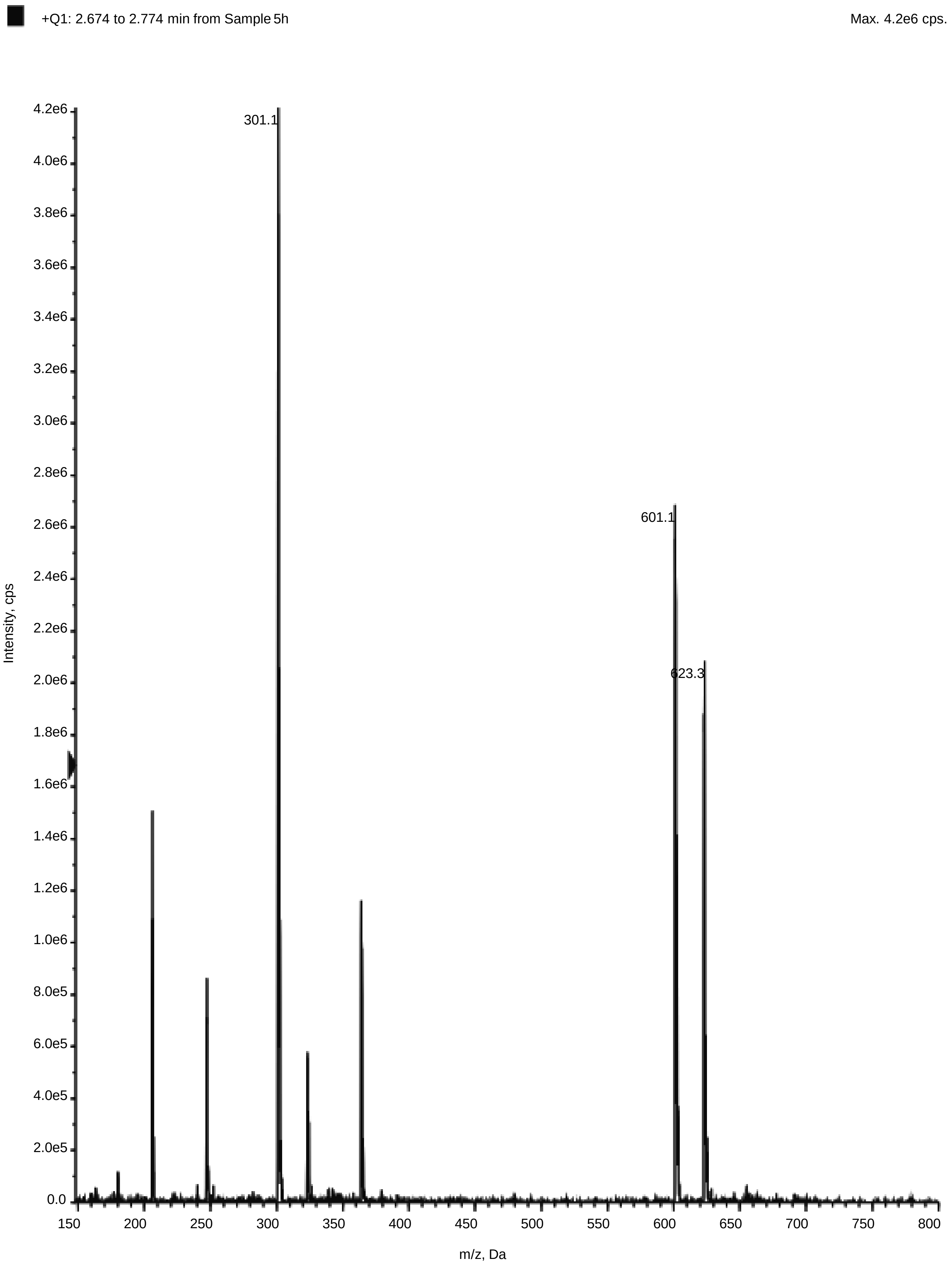

Figure S5 HNMR Spectrum of compound 5i in DMSO

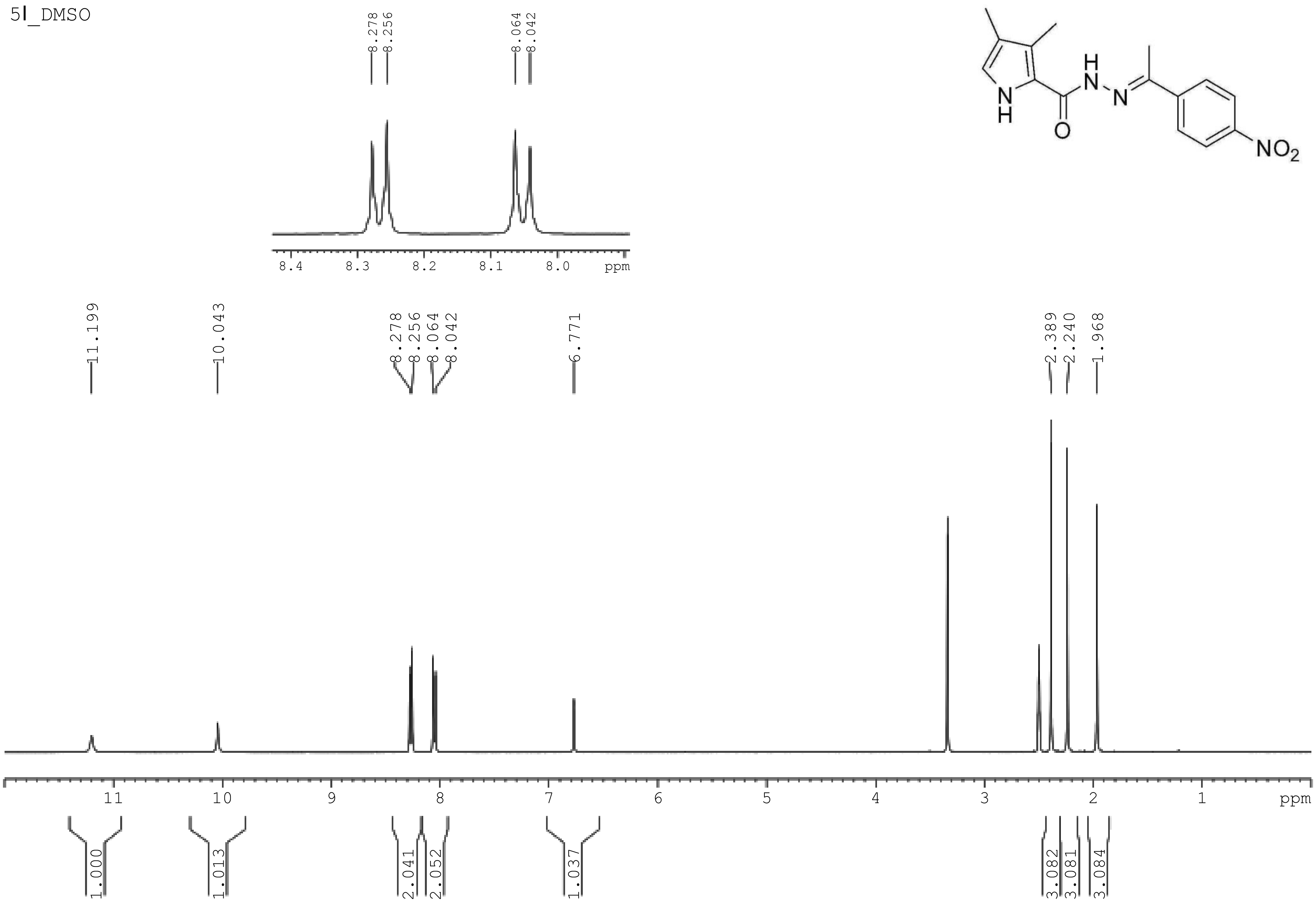

Figure S6 C13-NMR Spectrum of compound 5i in DMSO

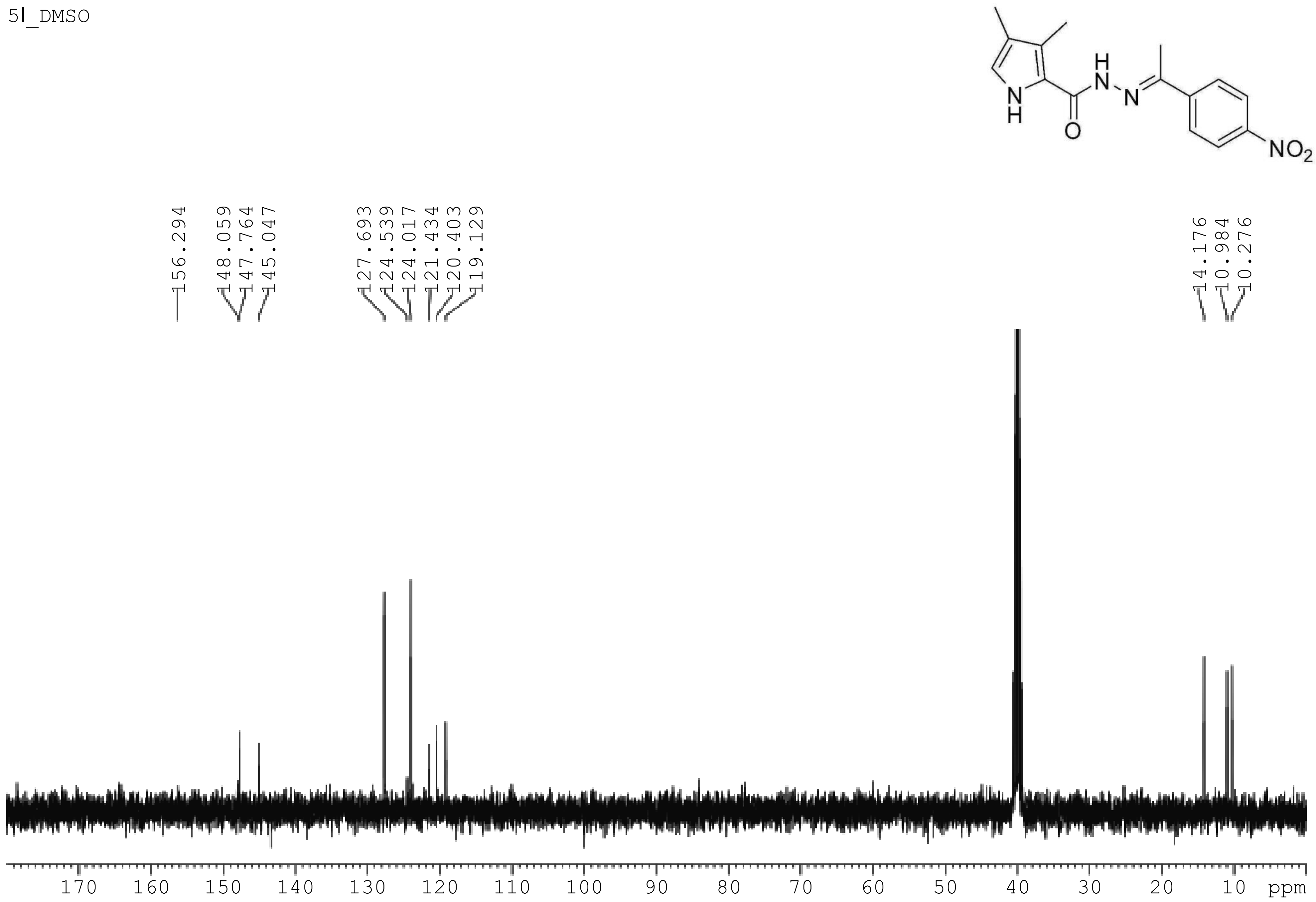

Figure S7 LCMS Spectrum with retention time (min) of compound 5i

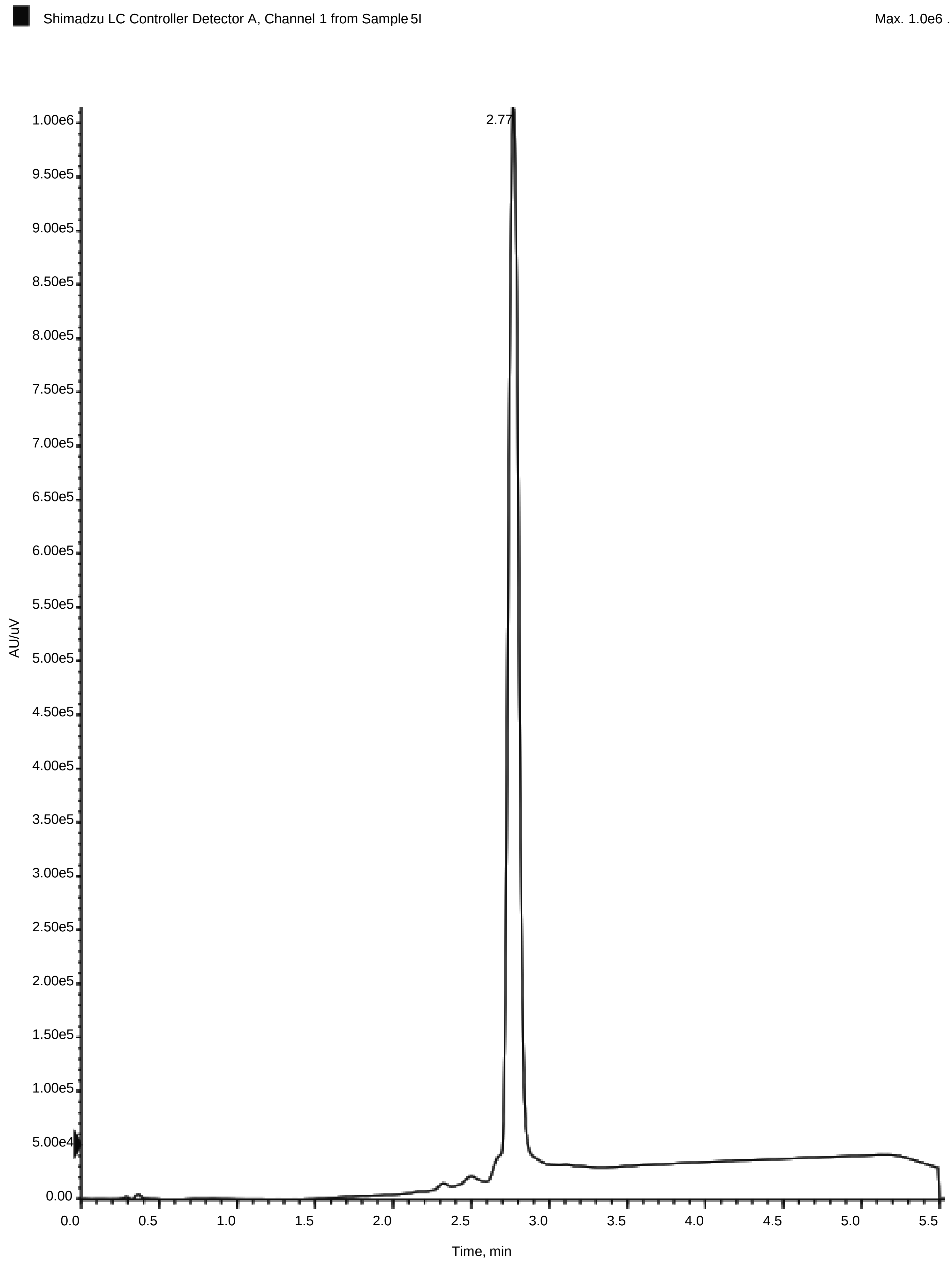

Figure S7 LCMS of compound 5i

Figure S8 MS Spectrum with retention time (min) of compound 5i

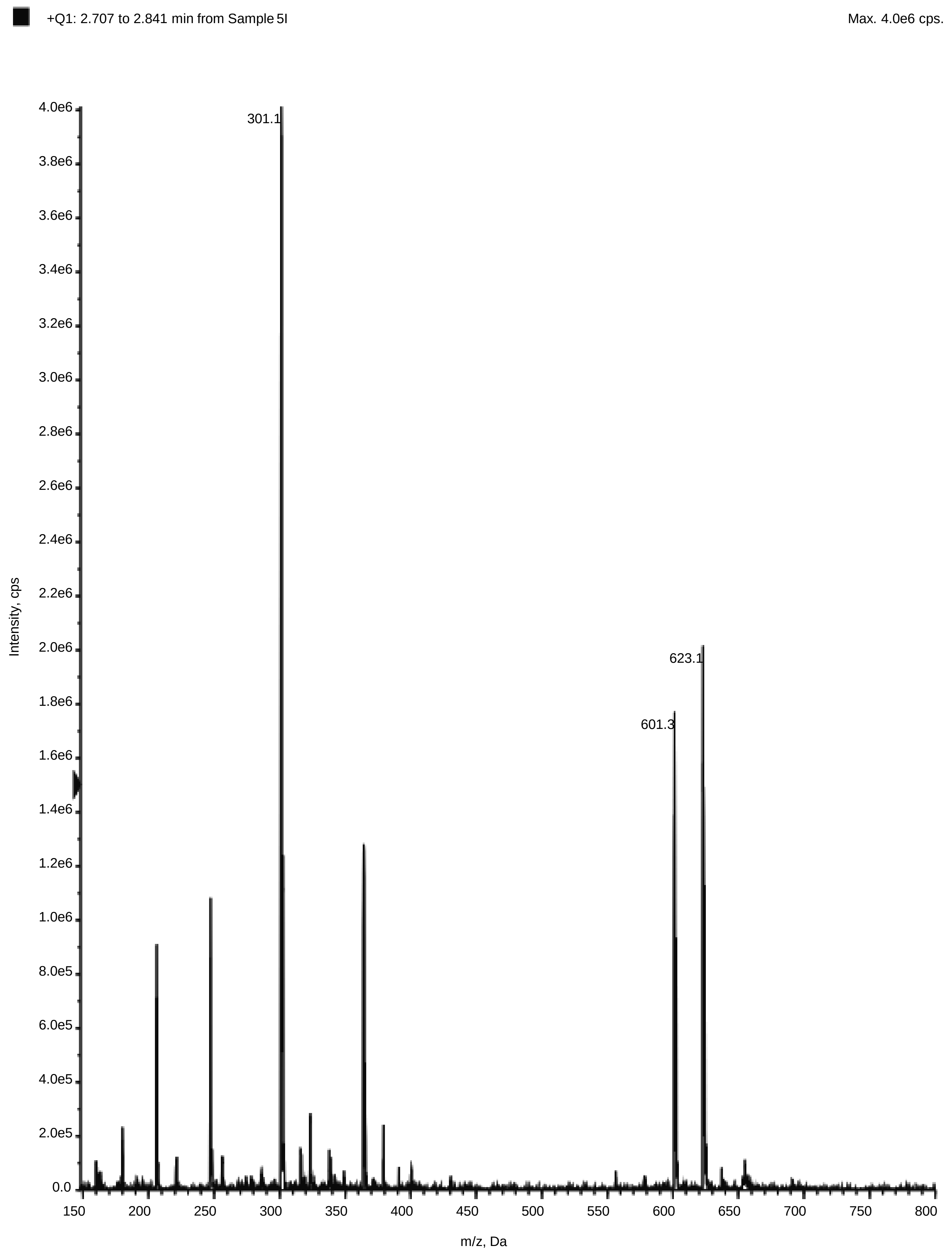

Figure S9 HNMR Spectrum of compound 5j in DMSO

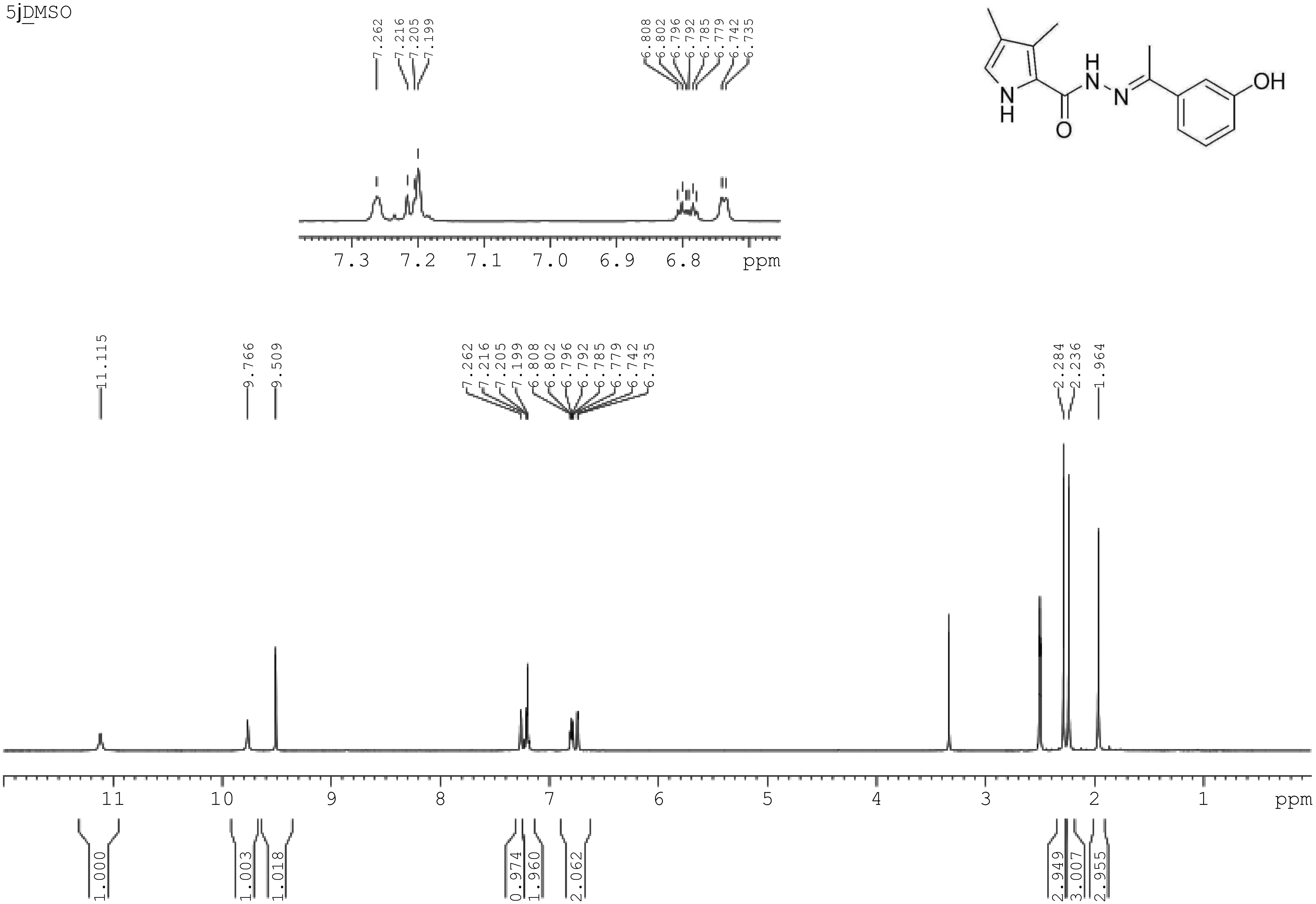

Figure S10 C13-NMR Spectrum of compound 5j in DMSO

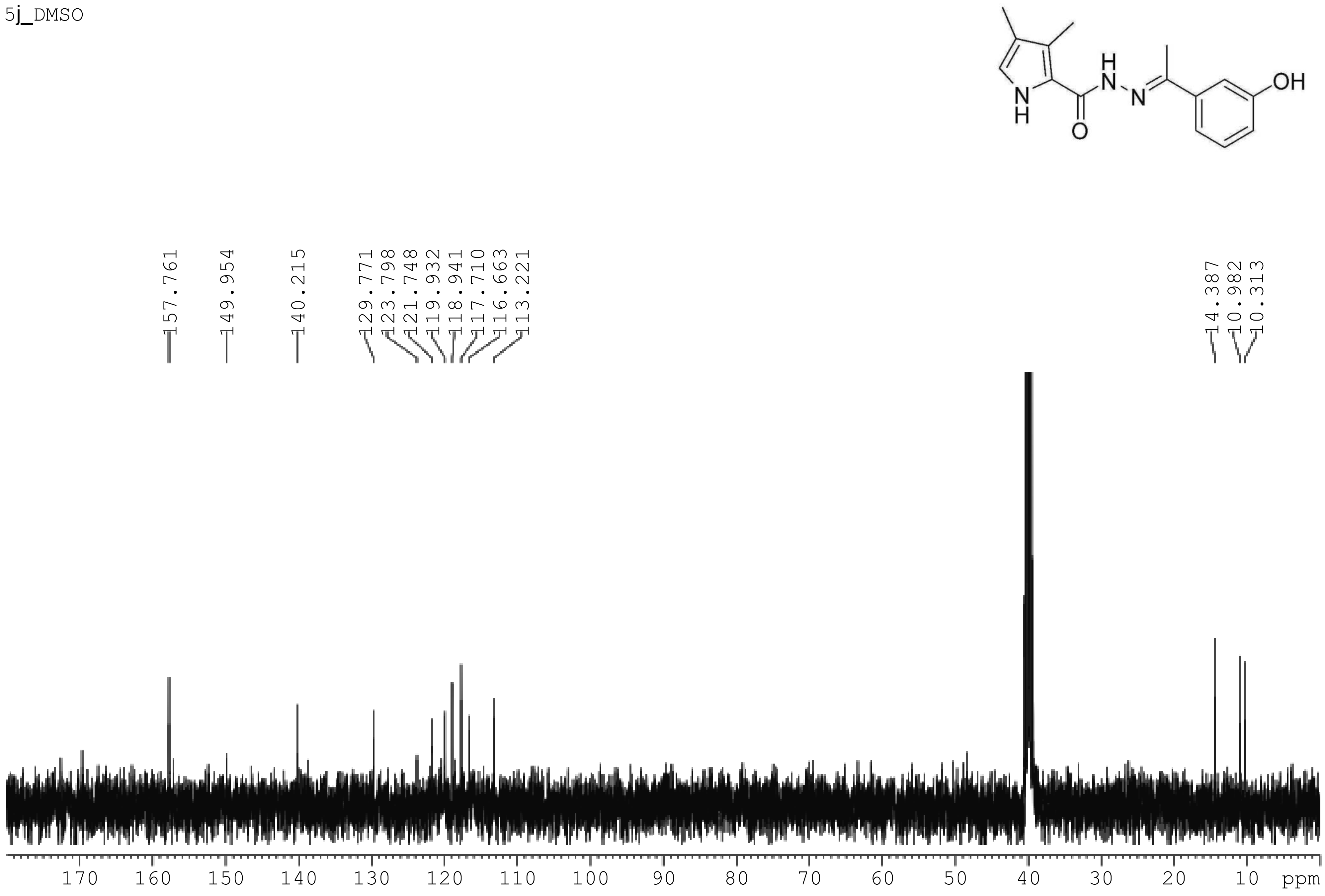

Figure S11 LCMS Spectrum with retention time (min) of compound 5j

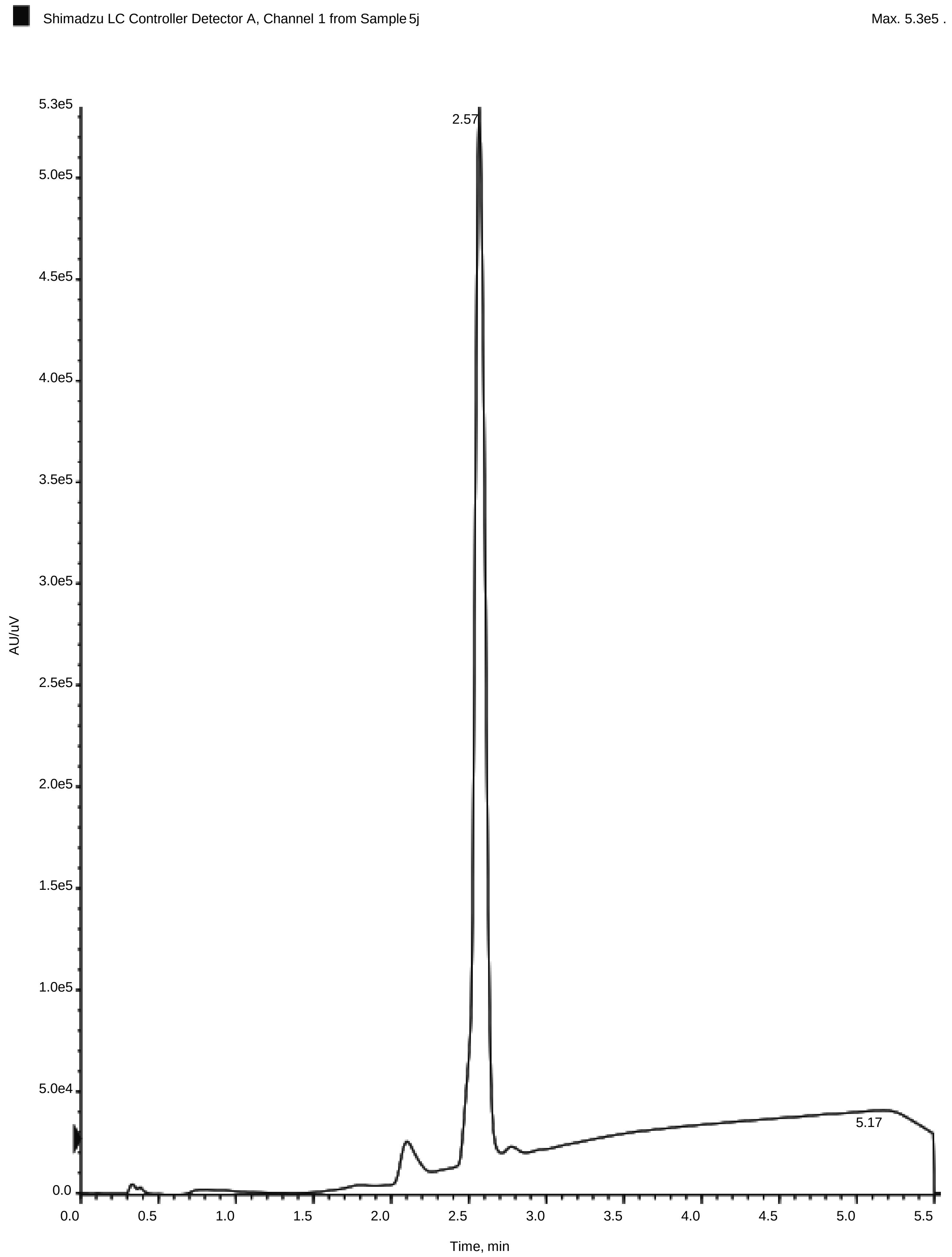

Figure S12 MS of compound 5j

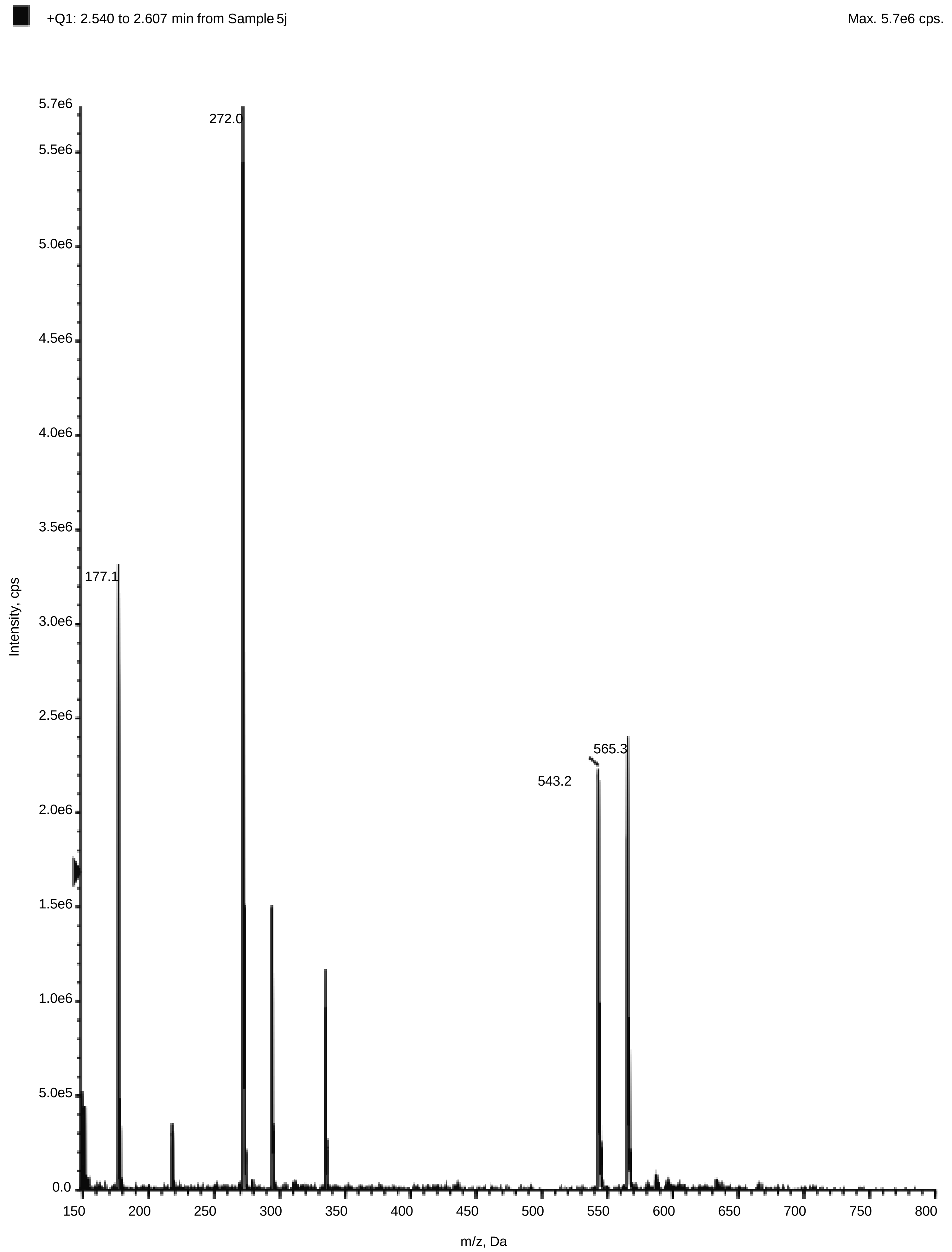

Figure S13 HNMR Spectrum of compound 3e in DMSO

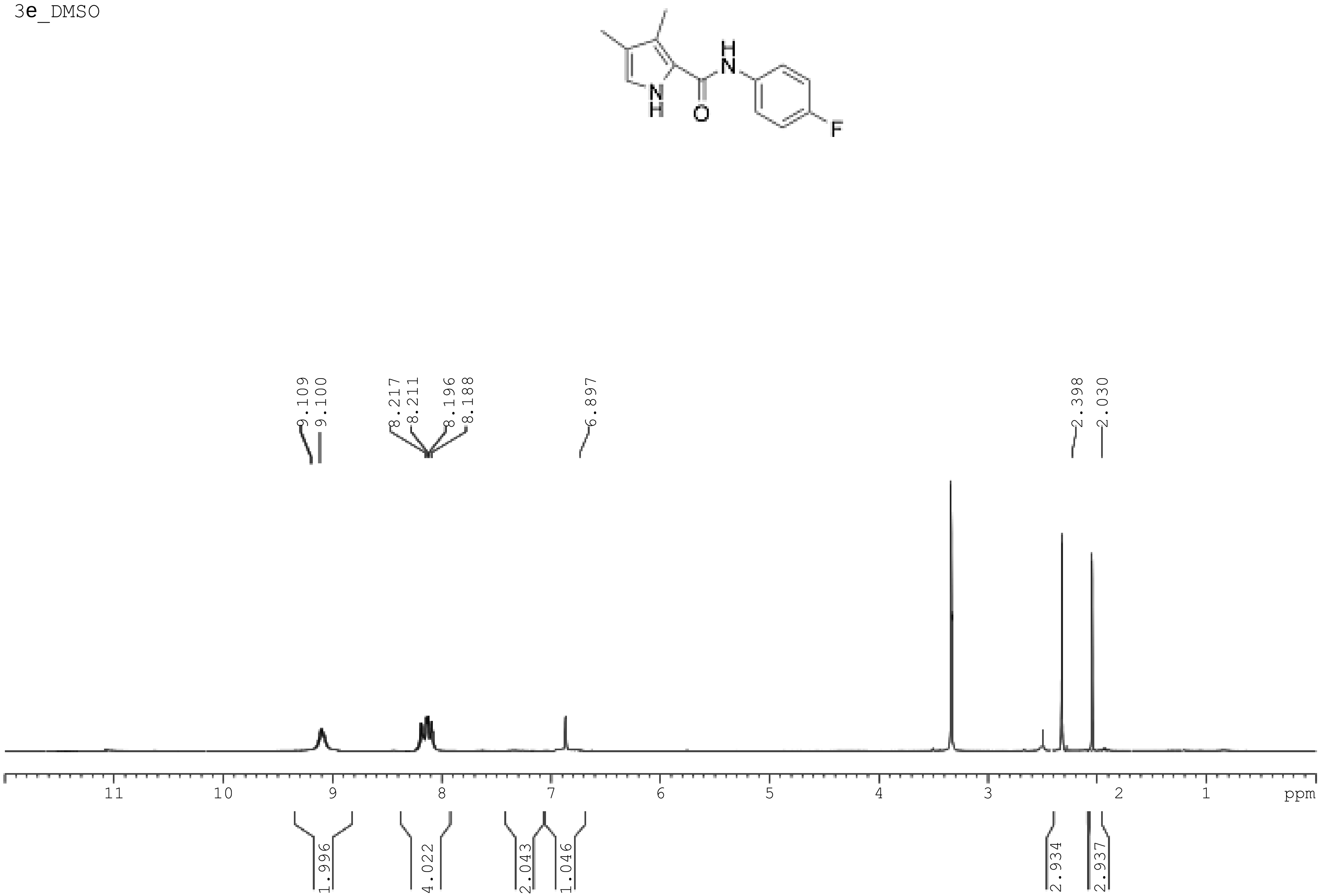

Figure S14 HNMR Spectrum of compound 3h in DMSO

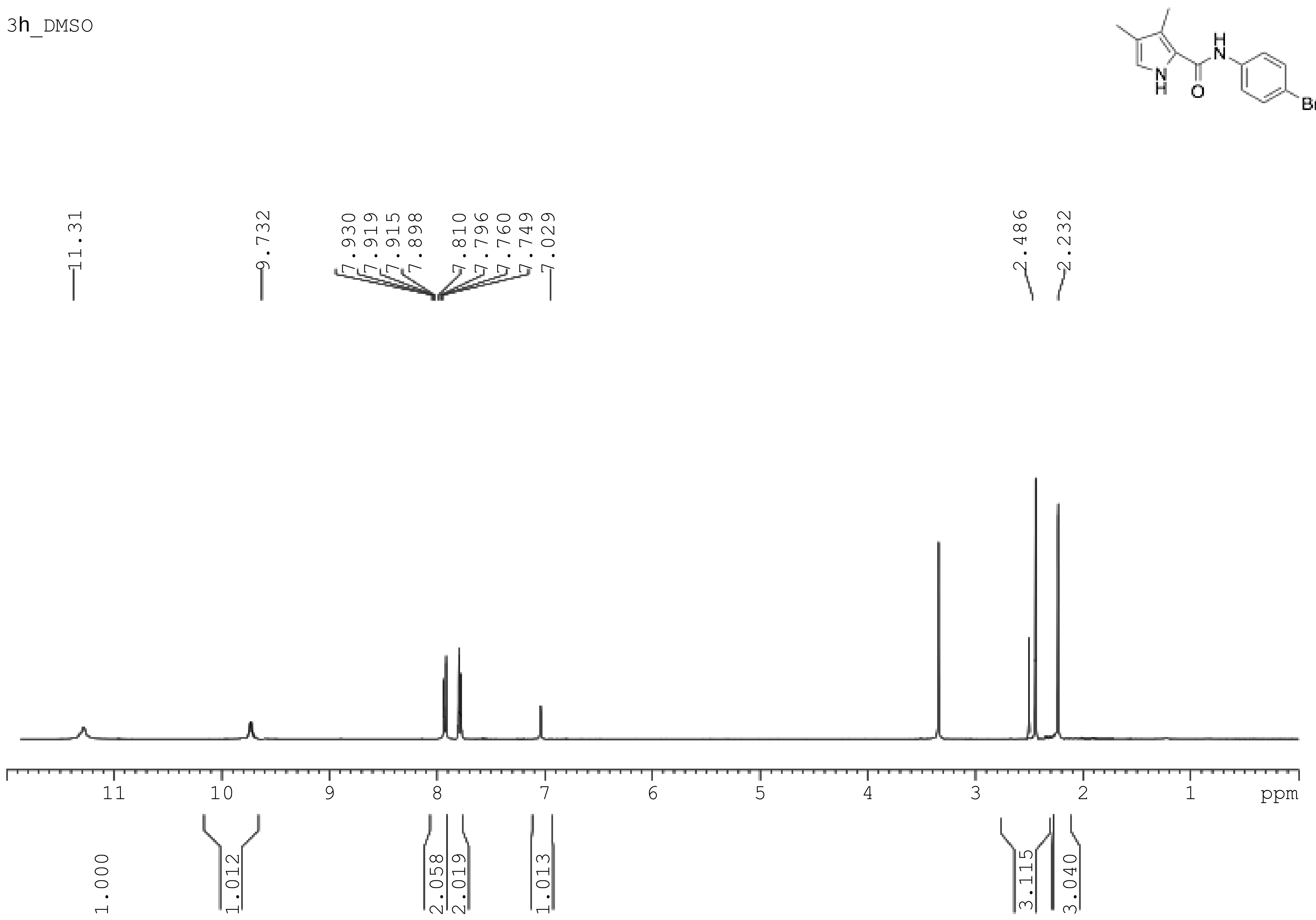

Figure S15 HNMR Spectrum of compound 3k in DMSO

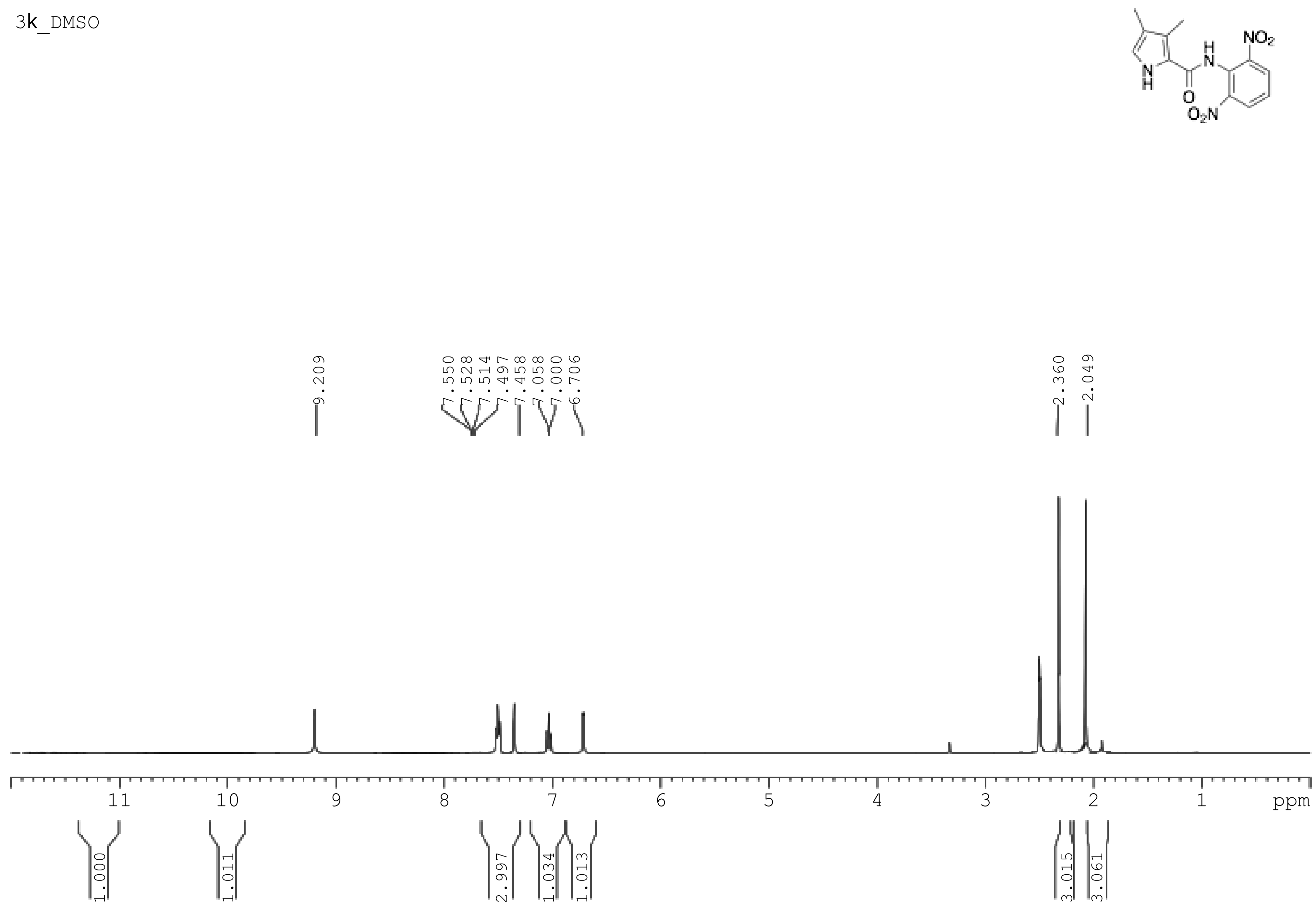

Supplement: Supplementary file 1 [file molecules-23-00875-s001.pdf]
